# Profile of osteopathic practice in Spain: results from a standardized data collection study

**DOI:** 10.1186/s12906-018-2190-0

**Published:** 2018-04-11

**Authors:** Gerard Alvarez Bustins, Pedro-Victor López Plaza, Sonia Roura Carvajal

**Affiliations:** 1 Centre for Osteopathic Medicine – C.O.ME. Collaboration, Spain National Center, Clinical-based Human Research Department, Barcelona, Spain; 2 Iberoamerican Cochrane Centre – Biomedical Research Institute Sant Pau, IIB Sant Pau, Barcelona, Spain; 3 Blanquerna School of Health Sciences, Ramon Llull University, Barcelona, Spain; 4 Fundació d’Osteopatia de Barcelona, Grup de Recerca en Osteopatia de Barcelona, Sant Just Desvern, Barcelona, Spain

**Keywords:** Osteopathy, Osteopathic medicine, Cross-sectional survey, Standardized data collection, Scope of practice, Clinical presentations

## Abstract

**Background:**

There is limited research regarding patients’ profiles and consumer attitudes and habits of osteopathy in Spain. The purpose of this study was to profile patients who regularly receive osteopathic care in Spain using an internationally developed standardized data collection tool.

**Method:**

During the period between April 2014 and December 2015, a UK-developed standardized data collection tool was distributed to Spanish osteopaths who voluntarily agreed to participate in this cross-sectional study.

**Results:**

Thirty-six osteopaths participated in this study and returned a total of 314 completed datasets. Of 314 patients, 61% were women and 39% were men, with a mean age of 40 years (SD 17.02 years, range 0 to 83 years). Forty-four percent were full-time salaried workers, and in 78% of cases, receiving osteopathic treatment was the patient’s own choice. Chronic spinal pain presentations were the most frequent reasons for consultation. Seventy-five percent of patients presented with a coexisting condition, mainly gastrointestinal disorders and headaches. The main treatment approach consisted of mobilization techniques, followed by soft tissue, cranial and high velocity thrust techniques. Improvement or resolution of the complaint was experienced by 93% of patients after a small number of sessions. Adverse events were minor and occurred in 7% of all cases.

**Conclusion:**

This is the first study carried out in Spain analyzing the profile of patients who receive osteopathic care. The typical patient who receives osteopathic care in Spain is middle-aged, presents mainly with chronic spinal pain, and voluntarily seeks osteopathic treatment. Osteopathic treatment produces a significant improvement in the majority of cases with a low rate of minor adverse events reported.

**Electronic supplementary material:**

The online version of this article (10.1186/s12906-018-2190-0) contains supplementary material, which is available to authorized users.

## Background

Osteopaths first started practicing in Spain in the 1980s. Since then, the practice of osteopathy in Spain has significantly developed. The first professional training and education programs in osteopathy began in 1987 and were taught by French and Belgian instructors [1]. Osteopathy is not regulated in Spain as a healthcare profession as it’s not included on the Law of Arrangement of the Sanitary Professions (LOPS 44/2003). In the other hand, the Ministerial Order (2135/2008) that establish the educational curriculum of the physiotherapy degree mention osteopathy as a technique that undergraduates shall know. However, both the standards and scope of practice of osteopathy in Spain lack formal recognition in the regulatory and legislative domains [2]. This situation has resulted in the co-existence of two recognized groups including ‘osteopaths’ (i.e., practitioners without prior health science degrees), and osteopath-physiotherapists (i.e., practitioners with prior health science degrees, typically in physiotherapy). Although the osteopath-physiotherapist group represents the majority of osteopaths practicing in Spain, there are other groups of practitioners with very diverse training and educational backgrounds. This ‘lawless’ situation has thereby fostered the emergence of numerous qualifications and professional associations representing different groups of osteopaths.

Additionally, there is limited research regarding the profiles of people who seek osteopathic treatment in Spain. In May 2008, the Observatory of Natural Therapies published an independent study, sponsored by three natural therapy organizations, on the use and habits of consumers of natural therapies in Spain [3] (Additional file 1). In this study, 2000 people aged between 16 and 65 years were interviewed. The results showed that osteopathy was known by 32% of the population and that 8.2% used it regularly. In 2011, the Spanish Ministry of Health, Social Policy and Equality published the report “Analysis of the situation of natural therapies” [4], which analyzed 139 techniques carried out within the natural therapies scope by assessing the existing scientific evidence, use and legal framework associated with these techniques in Spain and other countries. This report, which received expert contributions from several professional registers and organizations, profiled osteopathy as a complementary and alternative medicine and classified it under the category “Manipulative and body based therapies”; however, this report failed to provide additional data regarding natural therapy consumer use and habits beyond that submitted in 2008 by the Natural Therapies Observatory [3].

In 2009, the National Council for Osteopathic Research (NCOR) in the United Kingdom (UK) developed and piloted a Standardized Data Collection tool (SDC) to profile the demographics and clinical presentations of patients receiving osteopathic care [5]. The study characterized osteopathic practice in the UK to establish standards for audit activities, obtain relevant information for the profession and develop a resource for research purposes. The study demonstrated that the SDC tool was able to generate a substantial amount of high quality information and was suitable for widespread use. For example, it was able to provide information about patients’ demographic characteristics, presenting symptoms, patient management and treatment, and results obtained. In the last few years, some studies have been conducted in different countries to trace the profiles of both the professionals and patients who receive osteopathic care [6–10]. The SDC tool was used in some of these studies.

The primary objective of this study was to profile patients who regularly receive osteopathic care in Spain using the “SDC tool” developed by Fawkes and colleagues, which has been modified for a Spanish population. A secondary objective was to describe the professional profile of active osteopaths in Spain through a parallel survey designed specifically for this purpose.

## Methods

### Participants

During the period between April 2014 and December 2015, the SDC tool was distributed to all osteopaths who voluntarily agreed to participate in this cross-sectional study. The pragmatic eligibility criteria included any professional who named their practice as osteopathy. For participant recruitment, a three-step process was established. First, the cooperation of all associations, Registers and schools of osteopathy in Spain country was required. At the same time, the research team launched an internet-based information and awareness campaign on social networks (Twitter and Facebook) specially focused on Spanish osteopathic community groups. An explanatory video on how to participate and complete the form was also distributed (https://vimeo.com/105709291). Through these channels, a contact email address and a specific acceptation form to participate was provided. When acceptance forms were received, the research team provided osteopaths with a study information sheet and instructions to provide to their patients so that they would be fully informed about the purpose of the study (Additional file 2). Two reminders were scheduled for those osteopaths who did not return any completed survey after having agreed to participate. To maintain anonymity and confidentiality, each osteopath was allocated a unique ID code to which they could add a sequential code (01, 02.) for the patient identifier. The Institutional Review Board of Barcelona Osteopathic Foundation approved the study (FOB04140001). Informed consent was assumed by participation in the study.

### The questionnaire

Once authorized by NCOR, the extended version of the SDC tool was translated and cross-culturally adapted to Spanish. The translation process involved all members of the research team and was completed in 4 steps. i) forward translation into Spanish (PP) ii) backward translation into English verifying equivalency to the original meanings (GA & SR) iii) and piloting the questions on a sample of 5 osteopaths and iv) modification of the forward translation after pilot feedback (SR). The questionnaire was uploaded to an internet-based survey platform (SurveyMonkey® Europe – Dublin, Ireland) (Additional file 3).

The study form had three parts, those corresponding to the 1st and 2nd consultations and the last consultation in the data collection phase. The modified SDC Tool consisted of 47 items separated in different blocks that covered information about healthcare quality and treatment during the osteopathic intervention process.

I. Initial consultation for a new episode: Comprised of 23 questions related to the patient’s medical history, lifestyle, previous treatments, presence of pain, anatomical location of their symptoms and reasons for consulting an osteopath.
II. Management and Treatment: Comprised of 2 questions regarding the initial treatment and management of the patient and included treatment approaches used after the first consultation.
III. Information and consent: Comprised of 7 questions covering information regarding consent and its associated characteristics.
IV. Second Consultation: Comprised of 5 questions about the outcomes and approach of subsequent appointments regarding the treatment and the duration of the visit.
V. Last consultation for the treatment of the episode: Comprised of 10 questions related to the state of the patient at the end of the data collection period, total number of treatments received, and the evolution and quantification of the initial symptoms.

The symptom area(s) where the patient reported their complaints was considered the reason for consultation, and multiple sites were allowed. The osteopath chose a maximum of 4 symptom areas ranked by importance to the patient (considered as the clinical presentation). For pain reporting, a numerical rating scale (NRS) was used [11]. Patients were asked to range their pain in a 0–10 scale, with 0 representing the absence of pain and 10 the worst pain possible. A self-reported improvement was also recorded.

For those osteopaths who requested the survey in paper format, it was sent along with detailed return instructions and a prepaid envelope. Osteopaths recorded information from 10 new consecutive patients to avoid selection bias. In addition, the osteopaths received a second survey with 20 questions covering their professional profile. These 20 questions collected data among the following 3 blocks: pre-training, osteopathic studies, and workplace location and characteristics (Additional file 4).

### Data management and statistical analysis

Data obtained from the on-line survey were automatically exported to a spreadsheet (Microsoft Excel® version 15.21.1 – Microsoft Ibérica, Madrid -Spain). Data obtained in paper format were manually entered into the same spreadsheet by SR who was also responsible of the data analysis. All of the information collected (either from complete and incomplete forms) were analyzed. The responses were coded quantitatively to enable a simple descriptive statistical analysis. The total number of answers available for each question are reported in the following sections.

## Results

Of the 61 osteopaths who agreed to participate in the study, 36 returned the questionnaires from which a total of 314 datasets/patients were analyzed (8.7 patients for each osteopath on average). Eighty-three percent of the compiled questionnaires originated from the same region of the country (Catalonia). Two hundred forty-five patients completed treatment during the data collection period, and 75 (24%) did not finish the plan of care for unknown reasons. Among the three parts of the SDC tool, we collected information about the first visit from 314 patients, about the second visit from 266 (85%) patients and regarding the last visit from 248 (79%) patients. Concerning the quality of the responses, some of the forms were delivered incomplete (partially filled). Notwithstanding this, the unanswered questions did not follow a specific pattern that can suggest lack of interest or insufficient training methods.

### Patients’ demographic information

Patient demographic data are shown on Table 1. From 314 datasets, 61% (*n* = 192) were women versus 39% men (*n* = 122), and all participants had a mean age of 40 years (SD 17.02 years, range 0 to 83 years). The majority of patients (77%, *n* = 241) were adults (> 18 years), whereas 15% (*n* = 47) were children (< 18 years), of whom 10% (*n* = 15) were under the age of 1.

**Table 1:** Patients socio-demographic Characteristics

	%	n
Sex		314
Female	61%	192
Male	39%	122
Age (years)		40 (mean)
Employment situation		314
Full-time employee	44%	137
Student	17%	54
Self-employed	14%	43
Retirement	8%	27
Unemployed	5%	16
Other (Employee temporary part-time, domestic employment, pensioner)	12%	37
Referral Source		313
Patient’s own choice	78%	244
Health care professional	21%	68
Other	1%	1
Previous Osteopathic treatment		313
No	73%	229
Yes	27%	84
Osteopathic reason for consultation		
Recommendation or Reference	75%	235
Previous unsuccessful treatment	34%	108
Their choice to receive osteopathic treatment	30%	93
Treatment options other than medication	26%	81
Manual treatment search	20%	64
Personal search	16%	49
Previous Osteopathic treatment experience	10%	31
Alternative to surgery	5%	16
Do not desire treatment programmes from Social Security services	4%	12
Awaiting for rehabilitation covered by the Social Security service	2%	7
Other	2%	7

Regarding the patient employment profile, 44% (*n* = 137) of the respondents were full-time salaried workers. In 78% (*n* = 244) of the cases, receiving osteopathic treatment was the patient’s own choice. The findings of this study reflected that the remaining 22% (*n* = 68) of referrals were provided by other health professionals including 23% from general practitioners, 18% from physiotherapists, 16% from orthopedic consultants, 7% from podiatrists and a minor percentage from other professionals such as gynecologists, optometrists, physical trainers, pediatricians, neurologists, psychologists, acupuncturists, osteopaths, internal medicine specialists, dentists and insurance companies. Notwithstanding this, the main source of referrals for patients was in most cases attributed to word-of-mouth recommendations (76%, *n* = 239). The patient paid out of pocket for treatment in 91% (*n* = 284) of the cases.

Seventy-three percent (*n* = 229) of the cases had not previously received osteopathic treatment, and the main reasons to initiate treatment were personal recommendation or direct referral (75%, *n* = 235), previous unsuccessful treatments (34%, *n* = 108) or an individual choice to receive osteopathic treatment (30%, *n* = 93) (Table 1). Eleven percent of patients reported that they had already received prior treatment for the same complaint; including chiropractic, massage, physiotherapy, acupuncture, Bach flower remedies, neural therapy, diathermy, podiatry, optometry, speech therapy and steroid injections.

The waiting time to be examined by an osteopath did not exceed one week as reported by 75% (*n* = 236) of patients. The remaining 25% (*n* = 76) had to wait more than 8 days. The median number of medical consultations patients had undergone before receiving osteopathic treatment was 2 visits.

### Reason for consultation (clinical presentation)

The three most frequent complaints reported were symptoms in the cervical area (20%) followed by the lumbar area (13%) and the head and facial area (13%) (Table 2). Eighty-three percent of patients included in the study expressed multiple complaints at their first consultation (55% presented two co-morbid conditions and 31% presented more than 3). Co-morbidities followed the same distribution and included cervical (25%), lumbar (18%) and head and facial area (11%) conditions.

**Table 2:** Reason for consultation (clinical presentation location) *n* = 311

	%
Neck-cervical	20%
Lumbar	13%
Head- Face areas	13%
SI/pelvis/groin	9%
Shoulder	7%
Knee	5%
Thoracic spine	5%
Chest, Rib cage	4%
Hip	4%
Foot	3%
Abdomen	3%
Ankle	3%
Gluteal region	3%
Elbow	2%
Hand	1%
TMJ	1%
Other	1%
Wrist	1%
Arm	1%
Muscle	0%
Calf	0%
Forearm	0%

*SI* Sacroiliac joint, *TMJ* Temporomandibular joint

Thirty-eight percent (*n* = 119) of patients reported suffering from their complaint for over a year, 9% (*n* = 27) between 6 and 12 months, 27% (*n* = 84) between 1 and 6 months and 27% (*n* = 83) less than one month. Forty-nine percent (*n* = 152) of patients reported that it was the first time they had suffered from their complaint, whereas 35% (*n* = 111) had experienced it on 4 or more occasions. Eighty-nine percent (*n* = 278) had not taken time off from work despite the problem. The mean value of the degree of pain reported at the start of treatment was of 6 points (SD 2.2) on the pain assessment scale (NRS).

Seventy-five percent of patients had medical history accounts (*n* = 234) vs. 25% (*n* = 79) without accounts. The most frequent medical history conditions were gastrointestinal (12%, *n* = 29), migraines (11%, *n* = 25), anxiety (9%, *n* = 22) and arterial hypertension (9%, *n* = 21).

### Therapeutic approaches

In 96% (*n* = 301) of patients included in the study, osteopathic treatment was considered appropriate. The most frequently used techniques during the first consultation were mobilization techniques (60%), soft tissue techniques (57%), high velocity technique (HVT - thrust techniques) (52%) and cranial techniques (46%). At the follow-up consultations, the most commonly used techniques were mobilization techniques (61%), cranial techniques (54%), soft tissue techniques (52%), and functional techniques (42%) (Table 3).

**Table 3:** Techniques Approaches

First Visit	*n* = 301	Follow-up visits	*n* = 266
mobilization techniques	60%	mobilization techniques	61%
Soft tissues	57%	Cranial Techniques	54%
HVT	52%	Soft tissues	52%
Cranial Techniques	46%	Functional Techniques	42%
Functional Techniques	38%	HVT	39%
Counseling on daily lifestyle and habits	31%	Visceral Techniques	29%
Visceral Techniques	28%	Counseling on daily lifestyle and habits	24%
Patient education	23%	Patient education	20%
Counterstrain	19%	Muscle Energy technique	12%
Muscle Energy technique	9%	Counterstrain	11%
Contraction/Relaxation/Stretching	8%	Contraction/Relaxation/Stretching	10%
Physical activity	7%	Relaxation techniques	7%
Other	6%	Physical activity	7%
Diet	4%	Other	6%
Acupuncture	3%	Diet	4%
Relaxation techniques	2%	Acupuncture	2%
Hands off	1%	Hands off	1%
Orthopedic equipment	1%	Orthopedic equipment	0%
Infiltration	0%	Infiltration	0%

*HVT* high velocity technique

The time spent on each treatment session was divided between visits of < 30 min, between 30 and 45, between 45 and 60 or > 60 min. The frequency of each session duration is shown in Table 4.

**Table 4:** Time spent in consultation

1^st^ Visit		*n* = 312	Successive visits		*n* = 263
Less than 30 minuts	1%	3	Less than 30 minuts	5%	14
Between 30 and 45 minuts	15%	47	Between 30 and 45 minuts	25%	67
Between 45 and 60 minuts	58%	180	Between 45 and 60 minuts	68%	179
More than 60 minuts	26%	82	More than 60 minuts	1%	3

In 80% of the cases, the therapists asked for patient consent before providing osteopathic treatment. Of the consent-based cases, 33% were performed verbally, 21% were written and 46% were both verbal and written.

In the majority of cases, patients were informed about the different treatment options (70%), the possible risks and side effects of the treatment (85%), the expected response to treatment (4%), the approximate number of sessions required (72%), ways to avoid relapse in the future (66%) and an explanation of the clinical problem they presented (97%). In most cases, patients received counseling on their lifestyle habits, diet, and physical activity (66%), and only in few cases (9%) were other aspects were recommended.

### Treatment results

After the first consultation, patients reported that they felt “much better” in 25% of cases, “improving” in 52% and “no better or worse” in 15%. At the end of treatment, 72% of patient datasets included in the study reported being “much better” or “better than ever”, as described in Table 5. The mean value on the NRS at the end of treatment was of 1.6 (SD 1.8) (Table 5).

**Table 5:** Results obtained & NRS scale

2^nd^ Visit results		*n* = 266	Last Visit results		*n* = 244
Better than ever	5%	13	Better than ever	17%	42
Much better	25%	66	Much better	55%	134
Improved	52%	137	Improved	21%	52
No better or worse	15%	43	No better or worse	6%	14
Worst	2%	5	Worst	0%	1
Far worse	1%	2	Far worse	0%	1
Worse than ever	0%	0	Worse than ever	0%	0
Average score on NRS scale					
Pretreatment					6
Post-treatment					1,6

Seventy-three percent (*n* = 194) of patients did not report any side effects to the treatment after the first visit, whereas the remaining 27% (*n* = 99) mainly experienced fatigue and / or increased pain-related side effects. At the end of treatment, 93% (*n* = 224) of patients did not have any adverse effects.

Among the 248 patients who finished their plan of care during the study period, the number of treatment sessions provided ranged between 1 and 3 in 57% of the cases, 3–6 in 39% of the cases, 6–9 in 2% of the cases and > 9 in 2% of the cases. The median number of consultations per patient was 3 consultations, and the treatment duration ranged from 1 to 3 months in 54% of cases, less than one month in 31% of cases and more than 4 months in 15% of cases.

Sixty-eight percent (*n* = 168) of patients completed treatment during the time period in which the study was conducted. Seventeen percent (*n* = 42) were still receiving osteopathic care upon completion of the study, and 15% (*n* = 38) abandoned or terminated their plan of care for various reasons (e.g., illness, funding problems).

Upon the patients’ last visits within the study period, 25% had completed the treatment plan, 35% voluntarily chose to return for a check-up, 17% continued treatment, and 23% were referred to some further diagnostic process or resumed previous treatments.

### Osteopaths demographics

The mean age of the osteopaths participating in the study was 36.6 years (SD 7.65 years, range 27 to 70 years), and all held university qualifications prior to their osteopathy studies. The demographic and academic characteristics of the osteopaths are shown in Table 6. In reference to the participants’ osteopathy education and training, 61% (*n* = 22) had more than 1500 h, 36% (*n* = 13) had between 1000 and 1500 training hours, and 3% (n = 1) had less than 500 h. Ninety-seven percent (*n* = 35) of the osteopaths worked in a private practice of which 71% (*n* = 25) did so in their own practice. Sixty-four percent (*n* = 23) worked with other osteopaths, and 61% (n = 22) were part of a multidisciplinary team. Eighty percent (*n* = 29) of osteopaths worked exclusively as an osteopath, and 20% (*n* = 7) of the therapists combined their activity with other professional activities. Ninety-four percent of the participants reported to treat patients with musculoskeletal complaints, 47% pediatric patients, 39% obstetric patients, 33% gynecological problems and 11% sport related injuries (Table 6).

**Table 6:** Therapists socio-demographic Characteristics

	%	n
Sex		36
Male	53%	19
Female	47%	17
Age (years)	36.7 (mean) - 7.65 SD	
Previous Studies	100%	
Physiotherapy	88.5%	31
Other	11.5%	5
School of Osteopathy		
Spanish	94%	34
Other	6%	2
Experience (years)	7 (mean) - 4.45 SD	
Type of patients		
Musculoskeletal	94%	34
Pediatrics	47%	17
Obstetrics	38.8%	14
Gynecologists	33.3%	12
Sports	11.1%	4

Fifty-five percent of osteopaths were registered to some osteopathic register or professional association. All of these osteopaths were from Registro de los Osteopatas Españoles (ROE) except one from the General Osteopathic Council (GOsC).

## Discussion

To the best of our knowledge, this is the first study attempting to describe the profile of osteopathic practice in Spain. The main aim was to determine not only the profile of patients who receive osteopathic care in Spain but also the main features of the service provided. Furthermore, it was of interest to record the profile of the professionals who participated in the study. The SDC tool offers extensive information about the patients’ demographic characteristics, their reported symptoms, the osteopathic management plan derived therefrom and the way in which the service is provided.

The socioeconomic profile of the patient seeking osteopathic care in Spain mostly corresponds to people (women> men) of middle age (mean = 40 years) who are full-time employees. These results agree with the only study previously published in Spain [3] and the studies by Burke et al. [6] and Fawkes et al. [7] performed in Australia and UK, respectively (Table 7). This profile has also been reported in patients who receive complementary and / or alternative medicines and other self-care activities [12, 13].

**Table 7:** Patients data compared to available literature data

	Cofenat et al. (2008) [3]	Burke et al. (2013) [6]	Fawkes et al. (2013) [7]	Wilkinson et al. (2015) [9]	van Dun et al. (2016) [10]	Alvarez Bustins et al. (2018) (current study)
Average age	36–45	30–50	44.8	40.3	45	40
Percentage of women	>Women	67.6%	56%	63%		61%
Percentage of men	<Men	32.4%	43%	37%		39%
Reason for consultation	–	Lumbar, cervical, EEII	Lumbar, cervical, pelvis	Lumbar, Head-area, facial, Cervical spine	Cervical and lumbar, headache, Cervical brachialgia	Cervical, lumbar, Head and facial area
Techniques used preferably	Acupuncture, yoga, Homeopathy, massage	Soft tissue, Muscle Energy technique, mobilization techniques, education	Soft tissue, mobilization techniques, HVT, education, cranial, Muscle Energy technique, functional	Cranial, soft tissue, mobilization techniques, functional, counterstrain,	Visceral, cranial	Mobilization techniques, soft tissue techniques, HVT, cranial techniques

In Spain, osteopathy is mainly provided within the private healthcare sector and generally is a health service not covered either by the National Health System or by most health insurance companies. It was noted that although the majority of patients were adults, 15% were patients under 14 years of age, showing the interest and use of parents in Complementary and Alternative Medicine (CAM) in Europe [14–17].

The results revealed that a large population of patients included in this study had never received osteopathic care. The proportion of referrals from other healthcare professionals was found to be small. The choice to seek and undergo osteopathic treatment is usually personal and pursued as an alternative to previous unsuccessful treatments. This finding is supported by research focusing specifically on CAM use by pain sufferers, which described patients’ opinions as dissatisfaction with their general practitioners’ (GPs) availability, wait time, or lack of benefit from conventional medical treatments for back pain [13].

The main reason for receiving osteopathic care in Spain is related to musculoskeletal problems, mainly in the cervical and lumbar spine. These outcomes were also noted in similar studies conducted in other countries [6–10].

The use of CAM for back pain was recently extensively evaluated by Murthy et al. [13] Osteopathy was among the 4 CAM modalities assessed in that study along with acupuncture, chiropractic and massage. According to Murthy and colleagues [13], the prevalence of osteopathic treatment for back pain ranged from 4.1 to 48.4% (mean: 17.3%; median: 8.4%) as reported by four population studies drawing on fieldwork with large samples.

Recent research on the prevalence of spinal pain in Spain demonstrated that, after a period of stability between the years 2004/5 and 2008/9 [18], there was increase in the prevalence during between 2008/9 and 2011/12, with values of 5.4% for neck pain and 8.56% for low back pain [19].According to Palacios-Ceña and colleagues [19], this increase can be partially explained by the economic crisis suffered in Spain in recent years. However, more than one-third of patients treated in the Spanish public Health System for non-specific low back pain continue to suffer from pain 2 months after their first visit and in up to 10% of these individuals, the pain becomes worse [20]. In addition, the probability of referral to physical therapy or rehabilitation in Spain is greater when low back pain is more intense (acute and subacute cases) [20]. Given this situation, along with the perception of the usefulness of manipulative therapies in the treatment of back pain [13, 21], it may explain why this distress is the main cause for seeking osteopathic care (both in Spain and in other countries), and moreover, most patients included in our study reported chronicity in their complaints. This finding is contrary to what Burke et al. [6] and Fawkes et al. [7] reported, as they noted that acute cases (less than 4–6 weeks) were mostly treated. This difference in the early care environment may be related to the lack of a specific regulation the osteopathic scope of practice within the Spanish population.

Despite spine-related clinical problems, patients with headaches, facial-related pain and other symptoms are commonly seen by osteopaths [9, 10]. In our survey, headaches were the 3rd most common reason for seeking osteopathic care (13%). Some studies have shown preliminary positive results about the effectiveness of osteopathic manipulative treatment (OMT) in migraine patients [22–25]. Moreover, there is some evidence that OMT may lower the cost of the treatment regimen for patients with migraine headaches [26]. Notwithstanding this, according to the available literature, there is a low level of evidence that OMT is effective in the management of headache [27].

In our study, 69% of patients had previously visited a medical physician for the same complaint, with an average number of consultations before the start of the osteopathic treatment of 2.8. Actually, the pattern of CAM use for back pain supplementary to conventional care was evident across back pain–specific population studies from North America, Europe, and Australia, thereby suggesting that back pain sufferers did not choose CAM instead of conventional medicine [13].

The data obtained regarding the reduction of pain both after the first consultation and at the end of the treatment indicate that osteopathic care is a good approach to relieve patients’ pain. Although the effectiveness of osteopathy for musculoskeletal pain has been disputed [28], several studies have shown its effectiveness in patients with both acute and chronic lower back pain [29–32], neck pain [33] and in other clinical presentations [24, 27, 34–39]. It is also worth highlighting the low percentage of adverse effects reported by the patients (7%) at the end of treatment. These results reinforce those reported by Fawkes et al. [7] and Burke et al. [6] and other studies evaluating other manual therapies [40, 41] and are consistent with systematic reviews assessing the adverse effects of manual therapies [42].

The average number of visits per patient was 3.6 visits, plus in more than half of the cases, treatment did not exceed 2 months. In light of these results, osteopathic care appears to be effective over a short period of time and with a relatively low number of sessions. Despite these results, the clinical effectiveness and economic assessment of osteopathic care has not been established by our study. Furthermore, a lack of evidence in the literature showing the cost-effectiveness of osteopathic treatment remains [43, 44]. However, the results note a high degree of patient satisfaction with osteopathic treatment. Beyond clinical outcomes, patient-centered care, which is also a key aspect within the osteopathic approach, has been proven to be one of the most promising and effective scopes within health care [45–47] for its major relevance towards the established therapeutic relationship, application of a holistic approach to solve patients’ distress and tailoring of a patient’s treatment based on their context [48].

### Limitations

The main limitation of this study is the low response rate. Although a total of 61 professionals decided to participate, in only 59% (*n* = 36) of the cases were data collected. The great heterogeneity of professional profiles and the undefined legal situation of the profession in Spain leads to poor cohesion in this sector. Moreover, the absence of a single official institution representing the professionals’ interest and scope of practice, together with the differences among stakeholders, hinder the development of the profession and the performance of studies at population scale. Nonetheless, the significant number of patients included (*n* = 314) enables obtaining, at least, reliable associated indicators about the profile of patients treated with osteopathy in Spain.

Likewise, 83% of the datasets originated from a single region of the country (Catalonia) leading to a demographic bias of the results. Consequently, the data obtained on the professionals’ profile (secondary aim) may not be fully representative and should therefore be analyzed and interpreted with caution. Despite some unpublished studies addressing this issue [49–51], there is still a lack of evidence regarding the professional profile in Spain. A new study to identify the professional profiles of Spanish osteopaths is currently being conducted based on previous experiences in Benelux [10] and Italy (in process).

Some other limitations arise from the methodology used to obtain the data. The SDC tool was developed and piloted in the UK and was used as faithfully as possible to the original version. The choice of the SDC tool responds to the will of using a validated tool, however, the osteopathic scope of practice in the Spain is different from that of UK and some questions may have different interpretation in the Spanish context. Additionally, the translation process of the questionnaire did not involve professional translators and only included a small pilot sample for testing of the final translation.

Finally, practitioners, rather than patients, were responsible for the data collection. Although written specific instructions were given to osteopaths (Additional file 2), no in-person training or quality control checks were performed to assure that the forms were completed accurately. This could be a source of potential bias towards favorable outcomes rather than all outcomes. Moreover, specifically those answers concerning ethics, good practices and patients’ satisfaction should be considered under the light of potential reporting bias.

## Conclusion

This study describes the profile of patients who receive osteopathic care in Spain. By using a modified UK-developed SDC tool, data on the socio-demographic characteristics, population use habits, clinical characteristics and therapeutic experience of patients seeking osteopathic care in Spain were collected. Secondarily, information was obtained regarding the osteopathic management of patients and the way in which this service is provided. This is the first study conducted in Spain to analyze the profile of patients who receive osteopathic treatment. This information can help to increase public awareness of the profession, aid the decision-making process of patients regarding their care, and contribute to an understanding of the value that osteopathy may have in Spanish healthcare.

## Additional files

Additional file 1: **Supplementary file**. Observatorio de las Terapias Naturales. (PDF 293 kb)

Additional file 2: Instructions to inform patients clearly and comprehensively about the purpose of the study. (ZIP 166 kb)

Additional file 3: Extended version of the SDC Tool, which was translated and cross-culturally adapted into Spanish. (ZIP 384 kb)

Additional file 4: Professional survey. A Survey containing 20 questions covering osteopaths’ professional profile. (ZIP 172 kb)

## Abbreviations

CAM: Complementary and alternative medicines
GPs: General practitioners
HVT: High Velocity technique/s
NCOR: National Council of Osteopathic Research
NRS: Numerical rating scale
SDC tool: Standardized data collection tool
SI: Sacro-ilíac joint
TMJ: Temporo-mandibular joint
