# Supplementary material for: Profile of osteopathic practice in Spain: results from a standardized data collection study
Source: BMC Complement Altern Med. 2018 Apr 11;18:129. doi: 10.1186/s12906-018-2190-0 (PMC5896131; doi:10.1186/s12906-018-2190-0)
Supplement: Supplementary file 1 — Supplementary file. Observatorio de las Terapias Naturales. (PDF 293 kb) [file 12906_2018_2190_MOESM1_ESM.pdf]

OBSERVATORIO DE LAS TERAPIAS NATURALES

PRESENTACIÓN DEL PRIMER ESTUDIO SOBRE USO Y HÁBITOS DE CONSUMO DE LAS TERAPIAS NATURALES EN ESPAÑA

"Una realidad más importante de lo que creemos"

Mayo 2008

Presentación del primer estudio sobre uso y hábitos de consumo de las TN en España.
©2008 OBSERVATORIO DE LAS TERAPIAS NATURALES.
R.P.I.: M-003012 / 2008
Depósito Legal: M- 21038-2008

Ninguna parte de esta publicación, incluido el diseño de la cubierta puede ser reproducida, almacenada, transmitida o utilizada en manera alguna ni por ningún medio, ya se eléctrico, químico, mecánico, óptico, de grabación o electrográfico, sin el previo consentimiento por escrito de las tres entidades que lo publican: COFENAT, CONAMAD y TENACAT.

Índice

QUIÉN HA REALIZADO EL ESTUDIO	1	Pág. 5
MOTIVOS PARA LA REALIZACIÓN	2	Pág. 6
PRINCIPALES OBJETIVOS DEL ESTUDIO	3	Pág. 7
RESULTADOS DEL ESTUDIO CUANTITATIVO	4	Pág. 8
RESULTADOS DEL ESTUDIO CUALITATIVO	5	Pág. 15
CONCLUSIONES GENERALES	6	Pág. 21
PROMOTORES DEL ESTUDIO	7	Pág. 24
OBSERVATORIO DE LAS TERAPIAS NATURALES	8	Pág. 27

I QUIÉN HA REALIZADO EL ESTUDIO

Bajo el patrocinio de COFENAT (Federación Española de Terapias Naturales y No Convencionales) CONAMAD (Sociedad Cooperativa de Terapias Naturales) y TENACAT (Federación de Asociaciones de Profesionales de Terapias Naturales) se ha encargado al Instituto DYM la realización del "Primer Estudio Profesional sobre Uso y Hábitos de Consumo de las Terapias Naturales en España".

¡El Instituto DYM es una de las empresas más reconocidas en España en estudios de mercado y opinión. Fue fundado en 1962 y ha sido pionero en múltiples desarrollos metodológicos en el campo de la investigación de mercado. Igualmente es una de las empresas más grande independiente de España que ofrece todos los servicios y soluciones a los problemas de investigación.

2 MOTIVOS PARA LA REALIZACIÓN

Con la realización, por primera vez en España de un estudio profesional de estas características, el "Observatorio de las Terapias Naturales" pretende dar a conocer a la opinión pública los resultados del mismo para mostrar la realidad actual de las terapias naturales en España y de esta manera volver a introducir el debate sobre la necesidad de regular el sector de las terapias naturales en las agendas políticas y sociales, definitivamente.

3 PRINCIPALES OBJETIVOS DEL ESTUDIO

Estudio Cuantitativo:

- Evidenciar el grado de conocimiento de las terapias naturales entre la población.
- Que terapias naturales ha utilizado en alguna ocasión.
- Grado de satisfacción.
- Terapia natural utilizada en los últimos 12 meses.

Estudio Cualitativo:

- Conocer que percepción tiene la sociedad española de las terapias naturales, desde el punto de vista del usuario y del no usuario.
- Actitudes y motivaciones que genera en su uso.

4 RESULTADOS DEL ESTUDIO CUANTITATIVO

¿Cómo se ha realizado el estudio cuantitativo?

Se ha entrevistado una muestra de 2.000 individuos, en el hogar, de entre 16 y 65 años de la península e islas Baleares con un error muestral del +/-2,25% y un nivel de confianza del 95%, del 12 de noviembre al 5 de diciembre de 2007.

4 RESULTADOS DEL ESTUDIO CUANTITATIVO

Principales conclusiones:

- El 95,4% de la población española conoce alguna terapia natural.
- Las terapias más conocidas por los españoles son:

**Yoga**	(Te)	**87,7%**
**Acupuntura / M.T. Ch.**	(Te)	**81,8%**
**Tai Chi**	(Te)	**64,3%**
**Quiromasaje**	(Tc)	**58,6%**
**Homeopatía**	(Te)	**50,6%**
**Naturopatía / Naturoterapia**	(Tn)	**33,5%**
**Osteopatía**	(Tc)	**32,1%**
**Drenaje linfático**	(Tc)	**27,1%**
**Reflexología podal**	(Tc)	**26,7%**
**Shiatsu**	(Te)	**23,4%**
**Terapia floral**	(Tp)	**18,7%**
**Reiki**	(Te)	**16,7%**
**Kinesiología**	(Te)	**11,0%**

4 RESULTADOS DEL ESTUDIO CUANTITATIVO

Principales conclusiones

- De un modo espontáneo, las terapias más conocidas son:

**Acupuntura**	**14,0%**
**Yoga**	**12,6%**
**Homeopatía**	**8,7%**
**Masajes**	**5,6%**

- El **23,6%** de la población española, es decir **10.590.161 personas***, ha utilizado alguna vez las terapias naturales.

*Fuente propia: Calculada sobre INE Población a 1 de julio de 2007: 44.873.567

4 RESULTADOS DEL ESTUDIO CUANTITATIVO

Principales conclusiones:

- Las terapias más utilizadas en alguna ocasión por los españoles son:

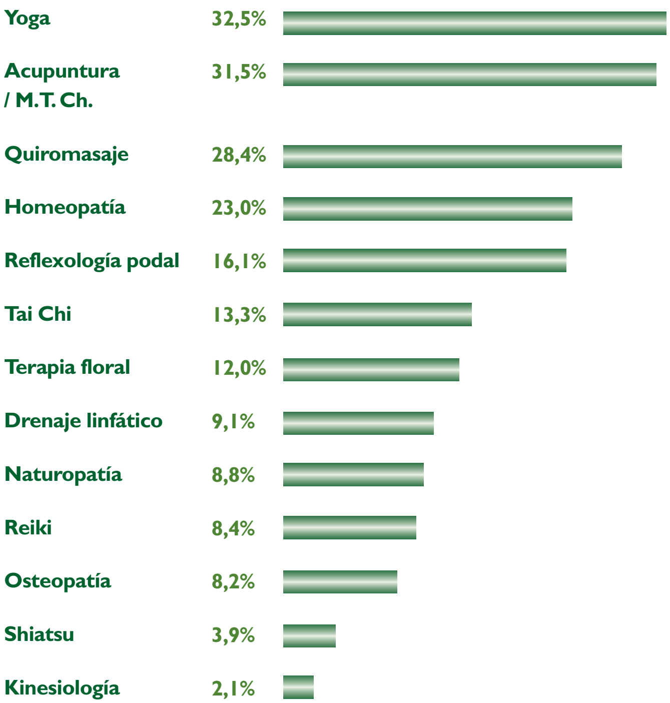

4 RESULTADOS DEL ESTUDIO CUANTITATIVO

Principales terapias utilizadas en alguna ocasión

Noroeste

Quiromasaje	6,9%
Yoga	6,9%
Acupuntura / M.T. Ch.	6,2%

Norte

Homeopatía	8,1%
Yoga	7,4%
Acupuntura / M.T. Ch.	7,3%

Noreste

Acupuntura / M.T. Ch.	12,5%
Quiromasaje	11,1%
Yoga	9,8%

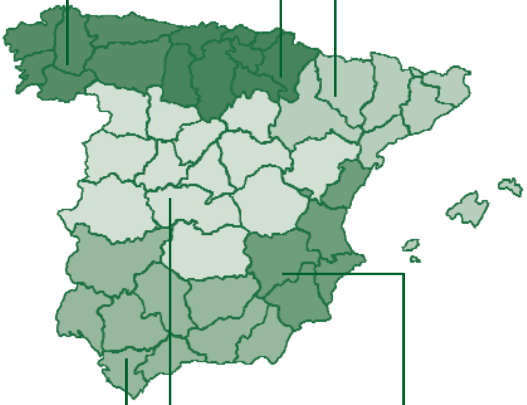

Sur

Acupuntura / M.T. Ch.	5,1%
Yoga	4,8%

Centro

Quiromasaje	10,6%
Yoga	6,3%

Levante

Yoga	7,5%
Quiromasaje	6,3%

Areas metropolitanas

Barcelona

Reflexología podal	16,9%
Homeopatía	15,2%
Acupuntura / M.T. Ch.	15%
Yoga	14,6%
Quiromasaje	10,8%
Terapia Floral	9,9%

Madrid

Yoga	8,1%
Acupuntura / M.T. Ch.	6,9%

4 RESULTADOS DEL ESTUDIO CUANTITATIVO

Principales terapias utilizadas en alguna ocasión por segmentos

Zona

Noroeste	21,4%	(213)
Norte	25,9%	(204)
Noreste	32,2%	(284)
Sur	15,2%	(404)
Centro	21,5%	(197)
Levante	24,4%	(297)
Barcelona A. M.	41,9%	(147)
Madrid A. M.	17,8%	(254)

Habitat

Areas metropol.	24,7%	(796)
de 30.000 a 200.000 hab.	21,9%	(442)
de 5.000 a 30.000 hab.	25,8%	(482)
menos de 5.000 hab.	19,4%	(280)

Clase social

Alta / Media-Alta	40,1%	(349)
Media	23,9%	(706)
Media-Baja	18,1%	(565)
Baja	15,9%	(380)

Edad

26 - 35 años	25,8%	(476)
36 - 45 años	30,7%	(410)
46 - 55 años	22,5%	(342)
56 - 65 años	20,6%	(291)

Sexo

Mujeres	26,7%	(1000)
Hombres	20,6%	(1000)

4 RESULTADOS DEL ESTUDIO CUANTITATIVO

Síntesis

• Perfil de los usuarios de las terapias naturales en España:

Mayor porcentaje de mujeres que hombres
Clase media alta / alta
Entre 36 y 45 años
Áreas metropolitanas.

• En el año 2007, un **12,9%** de la población española, es decir **5.788.690** personas han utilizado las terapias naturales.

•Grado de satisfacción con el uso de las terapias naturales:

4,18 sobre 5.

*Fuente propia: Calculada sobre INE Población a 1 de julio de 2007: 44.873.567

5 RESULTADOS DEL ESTUDIO CUALITATIVO

¿Cómo se ha realizado el estudio cualitativo?

Universo: Ha estado formado por público usuario y no usuario de terapias naturales.

Técnica: 5 Reuniones de Grupo que tuvieron una duración aproximada de 2 horas cada una de ellas, realizadas en Madrid y Barcelona.

Características Muestrales:

	**Edad**	**Tipos de Terapias**
**Público usuario**	Dos cortes de edad: de 18 a 35 años y de 36 a 60 años.	Representación de las principales terapias y con diferente nivel de antigüedad en su práctica.
**Público no usuario**	Un corte de edad: de 30 a 55 años.	Representación del conocimiento de algunas terapias.

5 RESULTADOS DEL ESTUDIO CUALITATIVO

Tendencia de las terapias naturales en los últimos años

- Se detecta una mentalidad más abierta, incluso en el colectivo sanitario.
- Aparición de nuevas terapias.
- Mayor interés y conocimiento por otras culturas.
- Mayor accesibilidad.
- Cubren necesidades derivadas del ritmo actual de vida.

Concepto de las terapias naturales

- Se relaciona con la salud física.
- Engloba la utilización de elementos y recursos naturales.

Modo de conocimiento

- El "boca-oreja" es la vía de conocimiento más habitual.
- A través de centros de salud natural, herbolarios, farmacias, etc. que actúan como fuentes de información.

5 RESULTADOS DEL ESTUDIO CUALITATIVO

Imagen de las terapias naturales

• ASPECTOS POSITIVOS

- Ofrecen un perfil positivo: eficacia, confianza, prestigio y seriedad.
- Tratan al ser humano desde un punto de vista holístico.
- Permiten la prevención y la curación (anímica y física).

• ASPECTOS A MEJORAR

- Desconocimiento.
- Informaciones incompletas y a veces confusas.
- Cierta desconfianza de algunas técnicas y de algunos profesionales.

Beneficios afectivo-emocionales de las terapias naturales

- Aumenta la comprensión y aceptación hacia uno mismo.
- Proporcionan paz, energía, tranquilidad, equilibrio y relajación.
- Propician un CAMBIO DE ESTILO DE VIDA.

5 RESULTADOS DEL ESTUDIO CUALITATIVO

Beneficios racionales de las terapias naturales

- Mejora física, ya que aprovecha los propios recursos sin efectos secundarios y son poco o nada agresivas.
- Son compatibles y complementarias a la medicina tradicional.

Aspectos afectivo-emocionales a mejorar de las terapias naturales

- Desconocimiento.
- Dudas.
- Cultura no occidental.

•Aspectos racionales a mejorar de las terapias naturales

- Profesionalización de centros.
- Equiparar el pago de los servicios al sistema sanitario convencional.

5 RESULTADOS DEL ESTUDIO CUALITATIVO

Importancia del profesional

- Es la premisa básica para la aceptación, confianza y credibilidad de las terapias naturales.
- Factores generadores de confianza:
  buena conexión empática con el cliente.
- Se le pide intuición, sensibilidad, capacidad perceptiva así como conocimientos teóricos y prácticos.
- Se le exige serenidad, tranquilidad, sosiego, equilibrio y capacidad para escuchar, saber expresarse y orientar.

Regulación del sector

- Una regulación oficial daría seguridad y confianza a los usuarios.

5 RESULTADOS DEL ESTUDIO CUALITATIVO

Toma de decisión

- ¿Por qué decide ACUDIR a las terapias naturales?
  - Tiene una motivación previa.
  - Tiene una mentalidad abierta.
  - Es la última alternativa que le queda ante diagnósticos y tratamientos fallidos.

¿Por qué decide NO ACUDIR a las terapias naturales?

- Por que no tiene la necesidad.
- Por falta de tiempo.
- Por incredulidad.
- Por el hecho de pagar.
- Por su orden de prioridades.

Nivel de satisfacción

- Satisfacción elevada.

6 CONCLUSIONES GENERALES

Situación general de las Terapias Naturales

• El dinamismo ha sido la tendencia imperante en el ámbito de las terapias naturales en los últimos años.

• La progresiva implantación de algunas terapias naturales, de forma complementaria a la medicina tradicional, en distintos países europeos y en EE.UU. y, más tímidamente en España, permite afirmar que existe un cambio en la manera de entender el cuidado de la propia salud.

• El concepto de terapias naturales presenta actualmente una imagen global beneficiosa tanto respecto al aspecto anímico-emocional como el físico para todos los segmentos analizados.

6 CONCLUSIONES GENERALES

Algunas dificultades presentes en las Terapias Naturales

- La dispersión de informaciones y estímulos que llegan al público, dificultan la permeabilidad de conceptos.

- Los profesionales no son siempre un soporte firme. Pueden presentar problemas de cualificación, credibilidad o praxis.

- Desde una vertiente individual, el perfil de imagen que se ha ido asentando sobre cada una de las terapias es desigual.

- Las variables que las segmentan inciden en los siguientes conceptos:

  Credibilidad, confianza,
  proximidad al concepto científico
  y consolidación en el mercado.

6 CONCLUSIONES GENERALES

Conclusiones finales

EL 95,4% DE LA POBLACIÓN ESPAÑOLA CONOCE ALGUNA TERAPIA NATURAL.

UNO DE CADA CUATRO ESPAÑOLES HA UTILIZADO ALGUNA VEZ LAS TERAPIAS NATURALES.

EL GRADO DE SATISFACCIÓN DE LOS ESPAÑOLES CON EL USO DE LAS TERAPIAS NATURALES ES CASI SOBRESALIENTE.

DURANTE EL AÑO 2007, CADA DÍA LABORABLE MÁS DE 26.300 ESPAÑOLES HAN UTILIZADO LAS TERAPIAS NATURALES.

7 PROMOTORES DEL ESTUDIO

COFENAT es una entidad asociativa y profesional de ámbito nacional inscrita en el Registro de Asociaciones sindicales y empresariales, que reúne a federaciones y asociaciones de profesionales de las terapias naturales de España.

COFENAT es la Sectorial de Terapias Naturales y UPTA España (Unión de Profesionales y Trabajadores Autónomos) y tienecomo objetivo fundamental la representación y defensa de los intereses de todos los profesionales que trabajan con métodos naturales de salud, actuales y tradicionales, y que ejerzan en el ámbito territorial del Estado español.

Presidenta: **Rafi Tur**

Teléfono de contacto: **607 946 787**

www.cofenat.es

cofenat
UPTA
FEDERACIÓN ESPAÑOLA DE TERAPIAS NATURALES Y NO CONVENCIONALES

7 PROMOTORES DEL ESTUDIO

TENACAT (Federación de Asociaciones de Profesionales deTerapias Naturales) es una federación de entidades profesionales de terapias naturales que mantiene la visión de promover el conocimiento de las terapias naturales dentro de la sociedad, facilitando su desarrollo con plenas garantías de calidad, a la vez que conservan sus filosofías y derechos de identidad.

Presidenta: **Francesca Simeón**

Teléfono de contacto: **616 237 461**

www.tenacat.org

tenacat Federación de Asociaciones de Profesionales de Terapias Naturales

7 PROMOTORES DEL ESTUDIO

CONAMAD (Sociedad Cooperativa de Terapias Naturales), es una cooperativa de trabajo asociado que agrupa a profesionales del ámbito de las terapias naturales y no convencionales con el objeto de poder prestar servicio de consulta, asesoramiento, formación, enseñanza y demás actividades económicas, agrupados en una estructura empresarial como marco, para defender sus intereses profesionales.

Presidente: Roberto Abitbol

Teléfono de contacto:
663 812 968

www.conamad.com

CONAMAD
Cooperativa Nacional de las Terapias Naturales

8 OBSERVATORIO DE LAS TERAPIAS NATURALES

Presentación

El Observatorio de las Terapias Naturales es una iniciativa de COFENAT, CONAMAD y TENACAT que está abierta a todas aquellas instituciones que se quieran sumar al proyecto.

El Observatorio de las Terapias Naturales es el resultado de la trayectoria profesional de un grupo de personas y de instituciones que creen en la necesidad de la existencia de un espacio abierto a todos los profesionales de las terapias naturales.

8 OBSERVATORIO DE LAS TERAPIAS NATURALES

Objetivo

El objetivo principal de este proyecto es crear una plataforma, como punto de encuentro permanente, dedicada a la divulgación, información y la formación en materia relacionada exclusivamente con las terapias naturales. Su marco de referencia territorial prioritario es España sin olvidar los estados europeos.

El Observatorio de las terapias naturales planifica sus actividades de manera interdisciplinar y quiere ser un lugar de encuentro de profesionales, instituciones, empresas y las diferentes administraciones públicas para aglutinar los esfuerzos de todos los colectivos que en España están vinculados al universo de las TT.NN. Igualmente se plantea ser un referente informativo para los medios de comunicación.

8 OBSERVATORIO DE LAS TERAPIAS NATURALES

Algunas de las actividades previstas en breve plazo son:

Investigaciones sobre la imagen, el uso y los hábitos de consumos de las TN en España.

Organizar actividades de interés para el sector.

Trabajar con las diferentes administraciones públicas para conseguir la regularización del sector de las terapias naturales.

Elaborar documentos de interés para el sector.

Confección de bases de datos y bibliográficas.

8 OBSERVATORIO DE LAS TERAPIAS NATURALES

Contacto

Director: **Alfons Vinyals**

Osteópata, Quiropractor y Homeópata en consulta privada, desde 1982.

Ha participado como docente desde 1985 en seminarios y talleres en postgrados en universidades y en convenciones y congresos.

Miembro de IFoH: International Federation of Heilpraktiker, desde 1990.

Obtiene en 1997 el "ACON Urkunde" diploma de reconocimiento internacional a la trayectoria profesional en Quiropráxia y Osteopatía.

Miembro del equipo profesional de la prueba piloto de evaluación de las terapias naturales en el hospital de Mataró.

Desde 2007 es titulado como experto universitario en Acupuntura Bioenergética por la USC (Universidad de Santiago de Compostela).

8 OBSERVATORIO DE LAS TERAPIAS NATURALES

Ha asumido los siguientes cargos y responsabilidades:

Secretario general 1985/1996 y Presidente 1996/2001 de APTI:
As. Prof. de Terapeutas Independientes.

Miembro de la Permanente de Sanidad del PSC-PSOE 1997.

Miembro de la Comisión de expertos para la regulación de las Terapias Naturales 1999/2007, siendo Experto asesor en terapias naturales en la Consellería de Salut de la Generalitat de Catalunya 2004/2007.

Secretario general de CTAC - UGT donde crea una sectorial de Terapias Naturales 2002/2005.

Presidente de la comisión gestora de TENACAT (Fed. de As. de Prof. de Terapias Naturales) 2005/2006.

Delegado - Coordinador de Salud de UPTA (Unión de profesionales y trabajadores autónomos) 2008.

Teléfono de contacto: **619 042 636**

Mail de contacto: **direccion@observatoriotn.org**
