# Supplementary material for: Profile of osteopathic practice in Spain: results from a standardized data collection study
Source: BMC Complement Altern Med. 2018 Apr 11;18:129. doi: 10.1186/s12906-018-2190-0 (PMC5896131; doi:10.1186/s12906-018-2190-0)
Supplement: Supplementary file 2 — Instructions to inform patients clearly and comprehensively about the purpose of the study. (ZIP 166 kb) [file 12906_2018_2190_MOESM2_ESM.zip › Additional file 1 (EN)R3.pdf]

Guide to complete the standardised data collection osteophatic tool (SCD modified).

Introduction

The standardised data collection tool is an instrument of capture of information and it has been developed by osteopaths in private practice in collaboration with NCOR (National Council for Osteopathic Research), to allow the collection of information which will help to profile both patients and approaches to practice. The SDC modified has been developed in the knowledge that osteopaths treat patients from a wide range of age groups and with an extensive array of symptoms. The information collected by the SDC will be helpful for individual clinicians, but also the profession as a whole as it deals with the demands of healthcare in the current century.

Ethical aspects

Ethical advice was sought early in the development of the SDC modified . All data collected will be anonymised and treated confidentially by the research team analysing the data. All the quiet information will be anonymous and treated of confidential form by the team of investigation for the analysis. Its permission was requested of form before to the patients for the securing, care and use of the information across the verbal request on the part of the osteopath.

The standardised data collection tool (SDC modified) is designed to facilitate the record of the information in the first visit and the follow-up of the patient in successive visits in practice clinic.

The document splits into 5 parts:

Part1 Contains the information of the patient related to the first visit.

Questions 6 and 7. It´s important to see the examples of playful or labor activities to indicate the most stated answer.

Question 12. How long does it have the patient to be waited to be visited?

This question refers to the waiting-list that the professional has well for the available hours in agenda, good as the only days used to clinic, good by the hourly availability of the patient.

Question 16. How long does the patient go with this problem (motive of consultation)?

This question refers to the duration in the time that the symptoms of the patient until he decides to look for treatment.

Question 21 To mark the severity of the main symptoms in the first visit

This visit is a modified visual analogical scale that tries to measure the gravity of the symptoms that the patient experiences. We have to explain to the patient the maximum and minimal value of the pain and symptoms and of subjective form to answer verbally and to mark.

Question 22 Anatomical areas of the symptoms:

It is important that the three main areas of symptoms are recorded. It is recognised that patients can consult for treatment with many more areas of pain or other symptoms. The categories have been modelled on those that appeared in two papers:

Parsons S, Carnes D, Pincus T, et al. Measuring troublesomeness of chronic pain by location. BMC Musculoskeletal Disorders. 2006;7:34.

Carnes D, Parsons S, Ashby D, et al. Chronic musculoskeletal pain rarely presents in a single body site: results from a UK population study. Rheumatology. 2007;46:1168.

Question a 23. Co-existing Conditions: Categories have been used that were published in:

Groll DL, To T, Bombardier C, et al. The development of a comorbidity index with physical function as the outcome. Journal of Clinical Epidemiology. 2005;58:595-602.

Part 2 Questions related to the management, the treatment and the advice to the patients.

Part 3 confidential dealing of the personal details and informed assent

(Parts 1,2 and 3 first visit)

Part 4 (the second visit) In this paragraph compilation of the information relative to the answer to the treatment after the first visit of the patient, and all the given advices and the process.

Question 33. Complications of the treatment: This question relates to findings documented in research studies including the work by Cagnie, Vinck, et al. looking at common side effects of manual treatments. This question specifically deals with any complications to treatment the patient experienced in the first 48 hours following the first treatment.

Cagnie B, Vinck E, Beernaert A, et al. How common are side effects of spinal manipulation and can these side effects be predicted? Manual Therapy. 2004;9:151-6.

Question 34 . Outcome of treatment

A number of rating scales exist, but many focus exclusively on pain. A generalised and validated global perceived effect scale, developed by Kemler et al. has been used in the SDC modified. This question specifically deals with the patient's overall outcome after their first treatment.

Kemler MA, De Vet HCW, Barendse H, et al. The effect of spinal cord stimulation in patients with chronic reflex sympathetic dystrophy: two years' follow up of the randomised controlled trial. Annals of Neurology. 2003;55(1):13-18.

Part 5 Last visit of the initial course of treatment for this episode

Question 41. Severity of the main symptoms. This question attempts to identify the severity of the symptoms present at the patient's last visit. This is a modified visual analog scale that tries to measure the severity of the main symptoms that the patient experiences. Is very important to explain the maximum and minimum value regarding the pain of their symptoms and subjectively answer verbally and mark it

Question 42 Complications of treatment. This question wants to know specifically if the patient is continuing to experience any lasting complications of treatment from the list provided. It is preferable if the patient completes this scale to avoid potential bias.

Question 43 . Outcome of treatment This question tries to identify specifically the patient's outcome of care at their final appointment or at their last appointment within the data collection period. It is preferable if the patient completes this scale to avoid potential bias.

Question 47 . What is the NATIONALITY of the patient? Ethnicity classifications have been taken from those recommended by the Equality and Human Rights Commission

http://www.equalityhumanrights.com/en/Pages/default.aspx.
This is a sensitive question for some patients and is optional if the patient feels uncomfortable providing this information.

Please, in case any of the questions is not clear enough, please let us know by email at the address we provide info@grostbcn.com.
