# Supplementary material for: Profile of osteopathic practice in Spain: results from a standardized data collection study
Source: BMC Complement Altern Med. 2018 Apr 11;18:129. doi: 10.1186/s12906-018-2190-0 (PMC5896131; doi:10.1186/s12906-018-2190-0)
Supplement: Supplementary file 2 — Instructions to inform patients clearly and comprehensively about the purpose of the study. (ZIP 166 kb) [file 12906_2018_2190_MOESM2_ESM.zip › Additional file 1R3.pdf]

Guía para completar la Herramienta de Recogida de Datos Osteopáticos Estandarizados (SCD modificado).

Introducción

La Herramienta para la recogida de Datos Osteopáticos Estandarizados es un instrumento de toma de información desarrollado por los osteópatas en práctica clínica privada en colaboración con NCOR (National Council for Osteopathic Research), cuyo objetivo es proporcionar información para crear un perfil de pacientes y enfoques para la práctica clínica de la profesión. Ha sido desarrollada con las condiciones en las que los osteópatas se encuentran cada día en consulta dentro de una amplio grupo poblacional, en edad y en diferentes síntomas.

La información recogida no sólo será importante para el colectivo médico/sanitario sino también para la profesión en su conjunto ya que conforma una necesidad asistencial social en el siglo actual.

Aspectos Éticos

Buscamos asesoramiento ético previo para conformación del formulario sometido a auditoria interna por el equipo de investigación. Todos los datos recogidos serán anónimos y tratados de forma confidencial por el equipo de investigación para el análisis de los mismos. Se solicitó de forma previa a los pacientes su permiso para la obtención, custodia y utilización de los datos a través de la petición verbal por parte del osteópata.

La Herramienta para la recogida de Datos Osteopáticos Estandarizados (SDC modificado) está diseñada para facilitar el registro de la información en la primera visita y el seguimiento del paciente en visitas sucesivas en la práctica clínica. Se divide en cinco partes :

Parte 1 contiene los datos del paciente en relación a la primera visita.

Preguntas 6 y 7. Ver los ejemplos de actividades lúdicas o laborales para señalar la respuesta más indicada.

Pregunta 12. ¿Cuánto tiempo tiene el paciente que esperar para ser visitado?

Esta pregunta hace referencia a la lista de espera que el profesional tiene bien por las horas disponibles en agenda, bien por los únicos días empleados a clínica, bien por la disponibilidad horaria del paciente.

Pregunta 16. ¿Cuánto tiempo lleva el paciente con este problema (motivo de consulta)? Esta pregunta se refiere a la duración en el tiempo que duran los síntomas del paciente hasta que decide buscar tratamiento.

Pregunta 21. Marcar la severidad de los síntomas en la primera visita Esta es una escala analógica visual modificada que intenta medir la gravedad de los síntomas que el paciente experimenta, explicar al paciente el valor máximo y mínimo respecto al dolor de sus síntomas y de forma subjetiva contestar verbalmente y marcar.

Pregunta 22. Áreas anatómicas de lo síntomas. Es importante que se registren las tres principales áreas en las que presenta los síntomas. Aunque el paciente puede consultar, como sabemos, por muchos otros síntomas y otras áreas de dolor. Las categorías que se han formulado están inspiradas en la literatura que se presenta a continuación:

Parsons S, Carnes D, Pincus T, et al. Measuring troublesomeness of chronic pain by location. BMC Musculoskeletal Disorders. 2006;7:34.

Carnes D, Parsons S, Ashby D, et al. Chronic musculoskeletal pain rarely presents in a single body site: results from a UK population study. Rheumatology. 2007;46:1168.

Pregunta 23. Antecedentes médicos. Categorías que se han utilizado que se publicaron en:

Groll DL, To T, Bombardier C, et al. The development of a comorbidity index with physical function as the outcome. Journal of Clinical Epidemiology. 2005;58:595-602.

Parte 2 recoge cuestiones relacionadas con la gestión, el tratamiento y el asesoramiento a los pacientes.

Parte 3 aborda el tema del consentimiento cuyos datos serán tratados de forma confidencial.

(Las Partes 1,2 y 3 forman parte de la primera visita)

Parte 4 *(referente a la segunda visita)* En este apartado se recoge la información relativa a la respuesta al tratamiento después de la primera visita del paciente , y todos los consejos dados y asesoramiento del proceso.

Pregunta 33. Las complicaciones del tratamiento: ¿Después de la primera visita el paciente comunicó alguna complicación del tratamiento en las primeras 48h? Esta pregunta se refiere a los hallazgos

documentados en estudios de investigación previos, referente a las posibles complicaciones posteriores al tratamiento durante las primeras 48 horas.

Cagnie, Vinck, et al. looking at common side effects of manual treatments.Cagnie B, Vinck E, Beernaert A, et al. How common are side effects of spinal manipulation and can these side effects be predicted? Manual Therapy. 2004;9:151-6.

Pregunta 34 . Resultado del tratamiento ¿Cuál fue el resultado general inmediatamente después de la primera visita? Muchas escalas se centran exclusivamente en el dolor, existe una escala validada que evalúa el efecto percibido tras el tratamiento desarrollada por Kemler et all utilizada en el formulario. Esta pregunta trata específicamente el resultado general del paciente después de su primer tratamiento.

Kemler MA, De Vet HCW, Barendse H, et al. The effect of spinal cord stimulation in patients with chronic reflex sympathetic dystrophy: two years' follow up of the randomised controlled trial. Annals of Neurology. 2003;55(1):13-18.

Parte 5 se refiere a la última visita del tratamiento para este mismo episodio.

Pregunta 41. Marcar la severidad de los síntomas en la última visita visita Esta es una escala analógica visual modificada que intenta medir la gravedad de los síntomas que el paciente experimenta, explicar al paciente el valor máximo y mínimo respecto al dolor de sus síntomas y de forma subjetiva contestar verbalmente y marcar.

Pregunta 42. Complicaciones del tratamiento ¿Continúa presentando el paciente alguna complicación (efecto secundario esperado) posterior al tratamiento? Esta pregunta trata de determinar específicamente si el paciente continúa experimentando cualquier complicación relativa al tratamiento recibido (según la lista proporcionada).

Pregunta 43 . Resultado del tratamiento ¿Cuál es la propia apreciación actual del paciente hasta la fecha? Esta pregunta trata de identificar específicamente los resultados de la atención del paciente en en su última visita en este periodo.

Pregunta 48 . ¿A qué Nacionalidad pertenece el paciente? La Clasificación étnica ha sido tomada del Informe de Igualdad y la Comisión de Derechos Humanos http://www.equalityhumanrights.com/en/Pages/default.aspx.

Esta pregunta puede resultar sensible para algunos pacientes y es opcional si el paciente se siente incómodo en proporcionar esta información o bien marcar si en la primera visita ya se ha indicado.

Por favor, en el caso de que alguna de las preguntas no sea lo suficientemente clara háganoslo saber y para agilizar el proceso recomendamos hacerlo por correo electrónico a la dirección que facilitamos info@grostbcn.com.

Final del proceso

Cuando haya completado todos los 10 formularios junto con el suyo como osteópata enviar a la dirección y sobre que facilitamos con los mismos. Gracias por participar en este estudio.

¿Qué sucede cuando finalizamos la recolección de datos?

El último día para la recogida de datos es ___________ .

Todos los formularios, los 10, serán devueltos y remitidos en el sobre que incorporamos en los formularios. Un informe final será enviado a cada profesional tras la elaboración del estudio y el análisis de los datos.

¿Qué hacer si necesita ayuda?

Si tiene algún problema durante el proceso o en la cumplimentación del formulario por favor contacte con nosotros a través del correo *info@grostbcn.es.*
