# Supplementary material for: Profile of osteopathic practice in Spain: results from a standardized data collection study
Source: BMC Complement Altern Med. 2018 Apr 11;18:129. doi: 10.1186/s12906-018-2190-0 (PMC5896131; doi:10.1186/s12906-018-2190-0)
Supplement: Supplementary file 3 — Extended version of the SDC Tool, which was translated and cross-culturally adapted into Spanish. (ZIP 384 kb) [file 12906_2018_2190_MOESM3_ESM.zip › Additional file 2 (EN)R3.pdf]

GROstBCN
Grup de Recerca en Osteopatia de Barcelona

THE STANDARDISED OSTEOPATHIC DATA COLLECTION TOOL

Osteopathic Standardised Data Collection(*)

This form is used in osteopathic practice in the USA and the UK to provide information about patients and treatments in each individual session. The osteopath must complete the form with a minimum of 10 patients and a maximum of 20. These patients must be consecutive and for the collection of data there is a section that must be completed during the first visit another in the second and a third at the end of process. The questions are translated into Spanish and are intended to be self- explanatory and unambiguous. Osteopaths should explain the questions of the form in an understandable way to the patients and the data will be collected and shared for anonymous and confidential study, so they must agree before starting the process.

Code ID del OSTEÓPATA: I__I__I__I__I__I__I Patient Number (01, 02, 03,…20): I__I__I
Example: Ramón Martínez Ramos: ramara)

Postal Code: I__I__I__I__I__I Data: I__I__I__I__I__I__I__I

I. INITIAL PRESENTATION FOR NEW EPISODE

1.Sex: Male ☐ Female ☐

2. Patient´s Age: I__I__I (years old)

3. What is the patient's main occupation?

____

No applicable ☐

4. What is the patient's current work status? (tick one as appropriate)

☐ Full time (employed)
☐ Full time (self-employed)
☐ Part-time (employed)
☐ Part-time (self-employed)
☐ Not currently employed
☐ Retired
☐ Student
☐ Pre-school
☐ Other

5. Does the patient receive disability allowance?

☐ Yes
☐ No
☐ Not applicable

6. How physically demanding is the patient's occupation? (**)

☐ strenuous
☐ sedentary
☐ moderate
☐ light
☐ not applicable

7. How strenuous are the patient's leisure time activities? (**)

☐ strenuous
☐ sedentary
☐ moderate
☐ not applicable
☐ light

(**) EXAMPLE LEISURE ACTIVITIES
Sedentary: handicrafts, cinema
Light: bad Light: minton, bowling, light gardening, walking (including to and from shops).
Moderate: jogging, swimming, moderate gardening.
Strenuous: basketball, competitive cycling, competitive swim

8. Who referred the patient to this practice?

☐ patient
☐ insurance company
☐ Social Security Consultant
☐ another healthcare practitioner
☐ GP
☐ employer
☐ solicitor

9. Has the patient ever had any osteopathic treatment before?

☐ Yes ☐ no

10. How did the patient hear about this practice? (tick all that apply)

☐ Word of mouth/recommendation
☐ Local advert
☐ Yellow pages
☐ I live nearby
☐ From a healthcare practitioner
☐ Internet search
☐ Other, please specify ______________________________

11. Why did the patient decide to have osteopathy? (tick all that apply)

☐ Personal recommendation or referral
☐ Personal research
☐ Waiting for NHS physio appointment
☐ Failure of previous treatment
☐ Previous experience of osteopathic treatment
☐ Desire to have osteopathic treatment
☐ Wanted a form of manual or hands on treatment
☐ Did not want treatment through the NHS
☐ Wanted to have drug-free treatment
☐ vOther, please specify ______________________

12. How long did the patient have to wait for the first appointment to be offered?

☐ Same día ☐ 2-3 days ☐ 4-7 days ☐ 8 days or more ☐ You don´t know

13. Is the patient on an Social Security waiting list for treatment for this condition?

☐ yes ☐ no

14. How long has the patient been waiting for Social Security treatment for this condition?

_____ days _____ Months No aplicable ☐

15. How many times has the patient visited their doctor about this condition prior to coming to here?___________ times

16. How many weeks has the patient had this current problem?

☐ Less than 1 week
☐ 1-2 weeks
☐ 3-4 weeks
☐ 5-6 weeks
☐ 7-12 weeks
☐ 13-51 weeks
☐ 1 year or more

17. How many weeks has the patient been off work with this current problem?

☐ less than 1 week
☐ 1 week
☐ 2 weeks
☐ 3 weeks
☐ 4 weeks
☐ 5 weeks or more
☐ not applicable

18. Has the patient had previous treatment or investigations for this episode of this condition?

Yes ☐ No ☐ If yes, has this included: Tick all that apply

	SS	Private
Imaging (XR, MRN,CTA,GMO,ECO)	☐	☐
Blood test	☐	☐
Medication	☐	☐
Urinalysis	☐	☐
Hospital outpatient treatment	☐	☐
Hospital inpatient treatment	☐	☐
Other (please state) ______________________	☐	☐

19. Type of onset of symptoms? Tick all that apply

☐ Acute/sudden onset (of unknown origin)
☐ Traumatic onset (of known origin)
☐ Slow/insidious onset
☐ Recurring problem

20. Is this the first episode? Please tick

☐ Yes, first time onset
☐ Second episode
☐ Third episode
☐ Fourth or more episodes

21. Severity of main symptoms on first visit – for patient completion

Best Imaginable 0 ☐----1 ☐----2 ☐----3 ☐----4 ☐----5 ☐----6 ☐----7 ☐----8 ☐----9 ☐----10 ☐ worse Imaginable

Moderate

22. Symptom areas: Please record up to four predominant symptom areas in order of priority for the patient

1st I__I__I 2nd I__I__I 3rd ☐ I__I__I 4th I__I__I

1 Head/facial area
2 Temporo-mandibular
3 Neck
4 Shoulder
5 Upper arm
6 Elbow
7 Forearm
8 Wrist
9 Hand 12 Lumbar
10 Thoracic spine
11 Rib cage
12 Lumbar
13 Sacroiliac/pelvis/groin
14 Gluteal region
15 Hip
16 Thigh/upper leg
17 Knee
18 Lower leg
19 Ankle
20 Foot
21 Abdomen
22 Other ____________________
____

23. What current co-existing conditions (diagnosed by a medical practitioner) does the patient have (tick all that apply)

☐ Anaemia
☐ Angina
☐ CHF (Congestive heart failure)
☐ Hypertension
☐ MI (myocardial infarct)
☐ Peripheral vascular disease
☐ Stroke/TIA (Transient Ischaemic Attack)
☐ Anxiety
☐ Depression
☐ Dementia
☐ Migraine
☐ Neurological disease
☐ Arthritis
☐ Asthma
☐ COPD (chronic obstructive pulmonary disease)
☐ Cancer
☐ Diabetes
☐ Hearing impairment
☐ Visual impairment
☐ Kidney disease
☐ Liver disease
☐ Pregnancy
☐ Bowel disease
☐ Upper gastrointestinal disease
☐ Other (please state)
☐ None

II. MANAGEMENT AND TREATMENT

24. What treatment plan was agreed with the patient?

- ☐ Osteopathic
- ☐ Non Osteopathic treatment
- ☐ Single consultation only
- ☐ Patient referred on ____________________

25. What types of treatment approaches have been used with the patient today?

- ☐ No hands on treatment
- ☐ Soft tissue
- ☐ Articulation
- ☐ HVLA thrust
- ☐ Cranial techniques
- ☐ Muscle energy
- ☐ Strain/counterstrain
- ☐ Functional technique
- ☐ Visceral
- ☐ Myofascial release (MFR)
- ☐ Education
- ☐ Relaxation advice
- ☐ Steroid Injection
- ☐ Acupuncture
- ☐ Dietary advice
- ☐ Exercise
- ☐ Orthotics
- ☐ Lifestyle advice
- ☐ Other (please name) _____________________

III. INFORMATION AND CONSENT (this information will be treated in strict confidence)

26. How was consent gained for examination?

- ☐ Implied consent
- ☐ Verbally
- ☐ Written
- ☐ Written and verbal
- ☐ Not applicable
- ☐ Other

27. Were any of the following procedures conducted and was specific consent obtained?

	CONDUCTED		CONSENTED		
	YES	NO	YES	NO	N/A
Per rectal	☐	☐	☐	☐	☐
Per Vaginal	☐	☐	☐	☐	☐
Oral	☐	☐	☐	☐	☐
Cervical HVT	☐	☐	☐	☐	☐
Lumbar HVT	☐	☐	☐	☐	☐
Toracic HVT	☐	☐	☐	☐	☐

28. Did you discuss with the patient

	YES	NO	N/A
Treatment options for their problem?	☐	☐	☐
Possible risks and side effects of treatment	☐	☐	☐
The anticipated response to treatment	☐	☐	☐
The anticipated number of treatments	☐	☐	☐
Ways to avoid recurrences in the future?	☐	☐	☐
An explanation of the presenting problem?	☐	☐	☐

29. What self-management strategies have been recommended for the patient to use?

☐ None
☐ Application of heat
☐ Application of cold
☐ Contrast bathing
☐ Rest
☐ Specific exercise
☐ General exercise
☐ Other (please state)
☐ Vitamins or other nutritional supplements
☐ Use of Back Book
☐ Use of Whiplash Book
☐ Natural remedies
☐ Naturopathic neuromuscular techniques
☐ Relaxation advice
☐ Advice concerning physical activity

30. Who is responsible for payment for treatment

☐ Self
☐ Insurance company
☐ Employer/own company
☐ Referral by doctor
☐ Other (please state)

31. Is an insurance case or litigation claim pending?

YES ☐ NO ☐

32. Time allocated for first appointment (minutes)

less than 30 ☐ between 30 and 45 ☐
between 45 and 60 ☐ more than 60 ☐

IV. SECOND APPOINTMENT

33. After the first appointment, did the patient report any complications of treatment within the first 48 hours?

☐ None of these
☐ Increased pain
☐ Increased stiffness
☐ Dizziness
☐ Nausea
☐ Headache
☐ Fatigue
☐ Serious adverse event, if known, please describe below

34. What was the patient's overall outcome after the first appointment?

☐ Worst ever
☐ Much worse
☐ Worse
☐ Not improved/not worse
☐ Improved
☐ Much improved
☐ Best ever

35. What types of treatment approaches have been used with the patient? Please tick all that apply

- ☐ No hands on treatment
- ☐ Soft tissue
- ☐ Articulation
- ☐ HVLA thrust
- ☐ Cranial techniques
- ☐ Muscle energy
- ☐ Strain/counterstrain
- ☐ Functional technique
- ☐ Visceral
- ☐ Myofascial release (MFR)
- ☐ Education
- ☐ Relaxation advice
- ☐ Steroid Injection
- ☐ Acupuncture
- ☐ Dietary advice
- ☐ Exercise
- ☐ Orthotics
- ☐ Lifestyle advice
- ☐ Other (please name) ____________________

36. What self-management strategies have been recommended for the patient to use? Please tick all that apply

- ☐ None
- ☐ Application of heat
- ☐ Application of cold
- ☐ Contrast bathing
- ☐ Rest
- ☐ Specific exercise
- ☐ General exercise
- ☐ Other(pleasestate)
- ☐ Vitamin or other nutritional supplements
- ☐ Use of the Back book
- ☐ Use of the Whiplash book
- ☐ Natural remedies
- ☐ Naturopathic neuromuscular techniques
- ☐ Relaxation advice
- ☐ Advice concerning physical activity

37. Time allocated for follow up appointments (minutes)

less than 30 ☐ between 30 and 45 ☐
between 45 and 60 ☐ more than 60 ☐

V. LAST VISIT OF INITIAL COURSE OF TREATMENT FOR THIS EPISODE

38. Date of final visit: I__I__I__I__I__I__I

39. Total number of treatments for this episodi to date: I__I__I

40. Has the patient completed the initial course of treatment for this episode?

- ☐ Yes
- ☐ NO, treatment is ongoing
- ☐ Patient did not return (reason unknown)
- ☐ Treatment terminated due to illness
- ☐ Treatment terminated due to finance
- ☐ Treatment terminated for other reason (please state)______________________________

41. Severity of main symptoms on last visit – for patient completion

0 1 2 3 4 5 6 7 8 9 10
Best Imaginable ☐----☐----☐----☐----☐----☐----☐----☐----☐----☐----☐ Worse Imaginable
Moderate

42. Is the patient continuing to report any complications of treatment

☐ None of these
☐ Increased pain
☐ Increased stiffness
☐ Dizziness
☐ Nausea
☐ Headache
☐ Fatigue
☐ Serious adverse event, if known, please describe

43. What was the patient's overall outcome at their final appointment or to date?

☐ Worst ever
☐ Much worse
☐ Worse¨ Not improved/not worse
☐ Improved
☐ Much improved ¨ Best ever

44. Did you contact the patient's doctor during this course of treatment?

☐ Yes ☐ No If yes, reasons for contact
☐ Patient was referred by the practice
☐ To request further information or investigation
☐ Other (please specify)
☐ GP had requested information
☐ To request referral for other treatment
☐ To provide the GP with information

45. At the last treatment, what was agreed for the patient's future care?

☐ None planned. Patient was discharged
☐ Patient opted to return for episodic care
☐ Patient awaiting results of investigation
☐ Patient was referred on for investigation/treatment
☐ Still continuing initial course of treatment
☐ Patient planning to return for further treatment
☐ Other (please state)

46. If the patient was referred for other treatment while still having osteopathic treatment, where were they referred to?

☐ Their Doctor
☐ Other medical consultant
☐ Other complementary practitioner ¨
☐ Physiotherapist or podiatrist
☐ A counsellor
☐ Exercise trainer or class
☐ Other (please state) ____________________

47. To which ethnic group does the patient belong? (this question is optional: the information is intended to try and serve all groups equally)

AREA OF ORIGIN

Latin America:
- ☐ ECUADOR
- ☐ COLOMBIA
- ☐ ARGENTINA
- ☐ DOMINICAN REPUBLIC ☐ BOLIVIA☐ PERU
- ☐ BRAZIL

Western Europe
- ☐ SPANISH
- ☐ UNITED KINGDOM
- ☐ GERMANY
- ☐ ITALY
- ☐ FRANCE
- ☐ PORTUGAL
- ☐ NETHERLANDS

Eastern Europe
- ☐ ROMANIA
- ☐ BULGARIA
- ☐ UKRAINE
- ☐ POLAND
- ☐ RUSSIA
- ☐ LITHUANIA

North Africa
- ☐ MOROCCO
- ☐ ALGERIA

Sub-Saharan Africa
- ☐ SENEGAL
- ☐ NIGERIA
- ☐ GAMBIA

Far East
- ☐ CHINA
- ☐ PHILIPPINES
- ☐ JAPAN

Indian Subcontinent
- ☐ PAKISTAN
- ☐ INDIA
- ☐ BANGLADÉS

North America
- ☐ USA

Middle East
- ☐IRAN
- ☐ SYRIA
- ☐ISRAEL

Oceania
- ☐ AUSTRALIA

Others and specify

____

Thank you for completing this form

Osteopathic Standardised Data Collection*

Statement of accreditation

"This standardised data collection tool has been produced by the National Council for Osteopathic Research (NCOR), and funded by the General Osteopathic Council (GOsC), the UK regulator of osteopaths. The intellectual property rights in the standardised data collection tool are jointly owned by the NCOR and the GOsC. The tool should be referenced in published work as: Moore AP, Leach CMJ, Fawkes CA. Standardised data collection tool for osteopathic practice. National Council for Osteopathic Research (UK) and General Osteopathic Council UK, 2009".
