# Supplementary material for: Profile of osteopathic practice in Spain: results from a standardized data collection study
Source: BMC Complement Altern Med. 2018 Apr 11;18:129. doi: 10.1186/s12906-018-2190-0 (PMC5896131; doi:10.1186/s12906-018-2190-0)
Supplement: Supplementary file 3 — Extended version of the SDC Tool, which was translated and cross-culturally adapted into Spanish. (ZIP 384 kb) [file 12906_2018_2190_MOESM3_ESM.zip › Additional file 2R3.pdf]

GROstBCN
Grup de Recerca en Osteopatia de Barcelona

# HERRAMIENTA PARA LA RECOGIDA DE DATOS OSTEOPÁTICOS ESTANDARIZADOS

*Osteopathic Standardised Data Collection(*)*

Este formulario se utiliza en la práctica osteopática en USA y en UK para proporcionar información sobre los pacientes y los tratamientos en cada sesión individual. Se solicita a cada osteópata completar el formulario a un mínimo de 10 pacientes y máximo de 20. Estos pacientes deben ser consecutivos y la recogida dispone de un apartado que debe cumplimentarse durante la primera visita, otro en la segunda y un tercero al finalizar el proceso. Las preguntas están traducidas al Castellano y están destinadas a ser autoexplicativas y sin ambigüedades. Los osteópatas deberán explicar de forma entendedora a los pacientes las preguntas del formulario, y que los datos serán recogidos y compartidos para estudio de forma anónima y confidencial, por lo que deberán estar de acuerdo antes de iniciar el proceso.

Código ID del OSTEÓPATA: I__I__I__I__I__I__I__I Número del paciente (01, 02, 03,…20): I__I__I
*Ejemplo: Ramón Martínez Ramos: ramara)*
I
Código Postal: I__I__I__I__I__I__I Fecha de la actuación: I__I__I__I__I__I__I__I

## I. CONSULTA INICIAL PARA UN NUEVO EPISODIO

1.Sexo del paciente: Masculino ☐ Femenino ☐

2. Edad del paciente: I__I__I (años)

3. ¿Cual es la principal ocupación del paciente?

______________________________________________

No aplicable ☐

4. ¿Cómo describiría la situación actual del paciente? (marque la que considere más apropiada)

- ☐ Trabajo a tiempo completo (empleado)
- ☐ Trabajo a tiempo completo (autónomo)
- ☐ Trabajo de media jornada (empleado)
- ☐ Trabajo de media jornada (autónomo)
- ☐ Actualmente en situación de desempleo
- ☐ Trabajo doméstico no remunerado
- ☐ Pensionista (Jubilación)
- ☐ Pensionista (Incapacidad)
- ☐ Estudiante
- ☐ Pre-escolar
- ☐ Otro (especificar)

5.¿El paciente recibe alguna pensión por discapacidad?

- ☐ Si
- ☐ No
- ☐ No aplicable

6. ¿Cómo es de exigente físicamente la profesión del paciente? (ver ejemplos de actividades)(**)

- ☐ sedentaria
- ☐ suave
- ☐ moderada
- ☐ extenuante
- ☐ no aplicable

7. Cómo de extenuantes/agotadoras son las actividades lúdicas del paciente? (ver ejemplos de actividades)(**)

- ☐ sedentaria
- ☐ suave
- ☐ moderada
- ☐ extenuante
- ☐ no aplicable

**(**)EJEMPLO DE ACTIVIDADES:** *Consideramos estas actividades tipo a señalar para valorar el grado de implicación activa y catalogar la actividad que realiza tanto desde el punto de vista lúdico como la implicación en el puesto de trabajo*
**Sedentarias**: cine, clases de dibujo *(lúdicas)* trabajo de despacho, recepcionista, taxista, conductor *(laborales)*
**Suaves**: caminar, ir de compras, jugar a los bolos *(lúdicas)* dependiente sin cargas, profesor *(laborales)*
**Moderadas**: natación, bici de paseo, trekking *(lúdicas)* manejo de cargas, operario *(laborales)*
**Extenuantes**: actividades deportivas dirigidas, competiciones deportivas, entrenamientos específicos *(lúdicas)* lampista, bombero, deportista de élite *(laborales).*

8. Quién deriva a nuestro paciente a la consulta?

- ☐ el propio paciente
- ☐ Médico de cabecera
- ☐ abogado
- ☐ una compañía aseguradora
- ☐ especialista de la Seguridad Social
- ☐ otro profesional de la salud

9. ¿Había recibido el paciente algún tratamiento osteopático con anterioridad?

☐ si ☐ no

10. ¿Cómo ha llegado el paciente a esta consulta? (señalar todas las que identifique)

- ☐ boca a boca/recomendado
- ☐ Anuncio local
- ☐ Páginas amarillas
- ☐ Domicilio cerca
- ☐ A través de un profesional de la salud
- ☐ Búsqueda por internet
- ☐ Otros (especificar)________________________

11. ¿Por qué el paciente decide hacer osteopatía? (señale todas las que identifique)

- ☐ Recomendación personal o referencias
- ☐ Búsqueda personal
- ☐ Esperando la rehabilitación de la Seguridad Social
- ☐ Tratamientos previos infructuosos
- ☐ Experiencias previas en tratamiento osteopático
- ☐ Deseo propio
- ☐ Búsqueda de un tratamiento manual
- ☐ No desea el tratamiento por Seguridad Social
- ☐ Quiere un tratamiento sin recurrir a la medicación
- ☐ Buscando una alternativa a la cirugía.
- ☐ Otros (especificar)________________________

12. ¿Cuánto tiempo ha tenido que esperar el paciente para ser visitado?

☐ Mismo día ☐ 2-3 días ☐ 4-7 días ☐ 8 días o más ☐ No se sabe

13. ¿Se encuentra el paciente en lista de espera para tratarse por la Seguridad Social para este problema (motivo de consulta)?

☐ si ☐ no

14. ¿Cuánto tiempo lleva de espera el paciente para tratarse por la Seguridad Social para este problema (motivo de consulta)?

_____ Días _____ Meses No aplicable

15. ¿Cuántas veces ha visitado al Médico antes de venir a consulta aquí? ____ veces

_____________________________________________________________________________especificar especialidad/des

16. ¿Cuánto tiempo lleva el paciente con este problema (motivo de consulta)?

- ☐ menos de 1 semana
- ☐ 1-2 semanas
- ☐ 3-4 semanas
- ☐ 5-6 semanas
- ☐ 7-12 semanas
- ☐ 4-5 meses
- ☐ 6-12 meses
- ☐ 1 año o más

17. ¿Cuánto tiempo lleva el paciente de baja laboral por este problema (motivo de consulta)?

- ☐ menos de 1 semana
- ☐ 1 semana
- ☐ 2 semanas
- ☐ 3 semanas
- ☐ 4 semanas
- ☐ 5 semanas o más
- ☐ no aplicable

18. El paciente se ha sometido ha alguna prueba o tratamiento con anterioridad por este episodio/problema/motivo de consulta?

Si ☐ No ☐ En caso afirmativo marcar, las que correspondan:

| | SS | Privado |
|---|---|---|
| Radiodiagnóstico (RX, RMN,TAC,GMO,ECO) | ☐ | ☐ |
| Análisis de Sangre | ☐ | ☐ |
| Medicación | ☐ | ☐ |
| Análisis de Orina | ☐ | ☐ |
| Tratamiento hospitalario ambulatorio | ☐ | ☐ |
| Tratamiento hospitalario (ingreso) | ☐ | ☐ |
| Otro (indicar cual) ___________________________ | ☐ | ☐ |

19. Tipo de inicio de los síntomas (Marcar todos los posibles).

☐ Agudo/Súbito
☐ Inicio traumático
☐ Progresivo/ insidioso
☐ Problema recurrente

20. ¿Es este el primer episodio que sufre?

☐ Si, la primera vez
☐ Segunda vez que sufre el mismo episodio
☐ Tercera vez que sufre el mismo episodio
☐ Cuatro o más episodios

21. Marcar la severidad de los síntomas en la primera visita *(Explicar al paciente los valores extremos para marcar el valor correspondiente al estado actual)*

0 1 2 3 4 5 6 7 8 9 10
Sin síntomas ☐----☐----☐----☐----☐----☐----☐----☐----☐----☐----☐ Lo peor, síntomas Inimaginable
Moderado

22. Áreas anatómicas de los síntomas: Marcar las áreas que el paciente identifique en orden de prioridad según sus síntomas.

1er I__I__I 2º I__I__I 3er ☐ I__I__I 4º I__I__I

| | | |
|---|---|---|
| 01 Cabeza/area facial | 09 Mano | 17 Rodilla |
| 02 Temporo-mandibular | 10 Columna torácica | 18 Pantorrilla |
| 03 Cuello/Cervical | 11 Tórax/caja torácica | 19 Tobillo |
| 04 Hombro | 12 Lumbares | 20 Pie |
| 05 Brazo | 13 Sacroiliaca/pelvis/ingle | 21 Abdomen |
| 06 Codo | 14 Región glútea | 22 Otros |
| 07 Antebrazo | 15 Cadera | ____________ |
| 08 Muñeca | 16 Muslo | ____________ |

23. Antecedentes médicos (con diagnóstico médico) :

☐ Anemia
☐ Angina
☐ Insuficiencia cardiaca
☐ Hipertensión arterial
☐ Infarto de miocardio
☐ Enfermedad Vascular periférica
☐ Accidente Cerebral Vascular
☐ Ansiedad
☐ Depresión
☐ Demencia
☐ Migrañas
☐ Enfermedad neurológica
☐ Artritis
☐ Osteoporosis
☐ Asma
☐ EPOC
☐ Cáncer
☐ Diabetes
☐ Pérdida auditiva
☐ Problemas visuales
☐ Enfermedad renal
☐ Enfermedad hepática
☐ Embarazo
☐ Enfermedad intestinal
☐ Enfermedad gastrointestinal
☐ Otras______________________________
☐ Ninguna

## II. GESTIÓN Y TRATAMIENTO

24. ¿Qué tipo de tratamiento se propone al paciente?

- ☐ Osteopatía
- ☐ Tratamiento NO Osteopático
- ☐ Simple consulta
- ☐ Se deriva al paciente a ____________________

25. ¿Qué técnicas de tratamiento se han realizado hoy para tratar al paciente?

- ☐ "hands off" (no tratamiento manual)
- ☐ Partes blandas/tratamiento tisular
- ☐ Articular
- ☐ Alta velocidad y baja amplitud
- ☐ Técnicas craneales
- ☐ Energía muscular
- ☐ Contracción/Relajación/Estiramiento
- ☐ Técnicas funcionales
- ☐ Técnicas viscerales
- ☐ Técnicas Miofasciales
- ☐ Educación al paciente
- ☐ Técnicas de relajación
- ☐ Infiltración
- ☐ Acupuntura
- ☐ Dieta
- ☐ Actividad física
- ☐ Material ortopédico
- ☐ Consejos, estilo y hábitos de vida
- ☐ Otros________________________

## III. INFORMACIÓN Y CONSENTIMIENTO (Esta información debe tratarse de forma confidencial)

26. ¿Cómo se obtuvo el consentimiento para el tratamiento?

- ☐ Consentimiento implícito
- ☐ Verbalmente
- ☐ Por escrito
- ☐ Por escrito y verbalmente
- ☐ No aplicable
- ☐ Otro

27. ¿Alguno de estos procedimientos fueron <u>indicados</u> y se obtuvo un <u>consentimiento</u> específico?

| | Indicados | | Consentimiento | | |
|---|---|---|---|---|---|
| | SI | NO | SI | NO | N/A |
| Tratamiento por vía rectal | ☐ | ☐ | ☐ | ☐ | ☐ |
| Tratamiento por vía vaginal | ☐ | ☐ | ☐ | ☐ | ☐ |
| Via intrabucal | ☐ | ☐ | ☐ | ☐ | ☐ |
| Manipulación Cervical | ☐ | ☐ | ☐ | ☐ | ☐ |
| Manipulación Lumbar | ☐ | ☐ | ☐ | ☐ | ☐ |
| Manipulación Torácica | ☐ | ☐ | ☐ | ☐ | ☐ |

28. Habló con el paciente para comentar (marcar todas)

| | SI | NO | N/A |
|---|---|---|---|
| Diferentes opciones para tratar su problema? | ☐ | ☐ | ☐ |
| Posibles riesgos y efectos secundarios del tratamiento? | ☐ | ☐ | ☐ |
| La respuesta esperada al tratamiento | ☐ | ☐ | ☐ |
| Número aproximado de sesiones necesarias | ☐ | ☐ | ☐ |
| Formas de evitar recaídas en un futuro? | ☐ | ☐ | ☐ |
| Una explicación del problema que presenta? | ☐ | ☐ | ☐ |

29. ¿Qué estrategias de autotratamiento se han establecido para el paciente durante el proceso?

- ☐ Ninguno
- ☐ Aplicación de calor
- ☐ Aplicación de frío
- ☐ Baños de contraste
- ☐ Reposo
- ☐ Ejercicio específico
- ☐ Ejercicio en general
- ☐ Otro (especificar) ___________________________
- ☐ Vitaminas y otros suplementos alimenticios
- ☐ Remedios naturales
- ☐ Técnicas neuromusculares en naturopatía
- ☐ Técnicas de relajación
- ☐ Consejos referentes a la actividad física

30. ¿Quién es el responsable del pago del tratamiento?

- ☐ El propio paciente
- ☐ Compañía de seguros (mútua)
- ☐ Prestaciones por empleado/propia empresa
- ☐ Otro (indicar)__________________________

31. Es un caso de litigio y lo lleva alguna compañía aseguradora, abogados, pendiente de juicio, forense…?

SI ☐ NO ☐

32. Tiempo asignado para la primera visita (minutos)

menos de 30 ☐ entre 30 y 45 ☐
entre 45 y 60 ☐ más de 60 ☐

## IV. SEGUNDA VISITA

33. ¿Después de la primera visita el paciente comunicó alguna complicación del tratamiento en las primeras 48h?

- ☐ No ninguna
- ☐ Aumento el dolor
- ☐ Aumento de la rigidez
- ☐ Inestabilidad, vértigos
- ☐ Nauseas
- ☐ Dolor de Cabeza
- ☐ Fatiga
- ☐ Estupor, somnolencia
- ☐ Efecto adverso severo (describir)___________

34. ¿Cuál fue el resultado general inmediatamente después de la primera visita?

- ☐ Peor que nunca
- ☐ Mucho peor
- ☐ Peor
- ☐ Ni mejor/ni peor
- ☐ Mejor
- ☐ Mucho mejor
- ☐ Mejor que nunca

35. ¿Qué enfoque/s de tratamiento/s se ha utilizado? Señale todos los utilizados

- ☐ "hands off" (no tratamiento manual)
- ☐ Partes blandas/tratamiento tisular
- ☐ Articular
- ☐ Alta velocidad y baja amplitud
- ☐ Técnicas craneales
- ☐ Energía muscular
- ☐ Contracción/Relajación/Estiramiento
- ☐ Técnicas funcionales
- ☐ Técnicas viscerales
- ☐ Técnicas Miofasciales
- ☐ Educación al paciente
- ☐ Técnicas de relajación
- ☐ Infiltración
- ☐ Acupuntura
- ☐ Dieta
- ☐ Actividad física
- ☐ Material ortopédico
- ☐ Consejos, estilo y hábitos de vida
- ☐ Otros________________________

36. ¿Qué estrategias de autotratamiento se han establecido para el paciente durante el proceso? Señalar todas las empleadas

- ☐ Ninguno
- ☐ Aplicación de calor
- ☐ Aplicación de frío
- ☐ Baños de contraste
- ☐ Reposo
- ☐ Ejercicio específico
- ☐ Ejercicio general
- ☐ Otro (especificar)____________________
- ☐ Vitaminas y suplementos nutricionales
- ☐ Remedios naturales
- ☐ Técnicas neuromusculates en naturopatía
- ☐ Técnicas de relajación
- ☐ Consejos referentes a la actividad física

37. Tiempo asignado para las visitas sucesivas:

menos de 30 ☐ entre 30 y 45 ☐
entre 45 y 60 ☐ más de 60 ☐

## V. ULTIMA VISITA DEL TRATAMIENTO PARA ESTE EPISODIO

38. Fecha final de la visita: I__I__I__I__I__I__I

39. Número total de tratamientos hasta la fecha, para este episodio: I__I__I

40. ¿Ha completado el paciente el tratamiento para este episodio (motivo de consulta)?

- ☐ SI
- ☐ NO, tratamiento en curso
- ☐ Abandono (razón desconocida)
- ☐ Finaliza tratamiento debido a una enfermedad
- ☐ Finaliza tratamiento debido a la financiación
- ☐ Finaliza tratamiento por otra razón (indicar)______________________________

41. Severidad de los síntomas principales en la última visita *(Explicar al paciente los valores extremos para marcar el valor correspondiente al estado actual)*

No síntomas 0 ☐---- 1 ☐---- 2 ☐---- 3 ☐---- 4 ☐---- 5 ☐---- 6 ☐---- 7 ☐---- 8 ☐---- 9 ☐---- 10 ☐ Lo peor de los síntomas inimaginable

Moderado

42. ¿Continúa presentando el paciente alguna complicación (efecto secundario esperado) posterior al tratamiento?

- ☐ No, ninguno
- ☐ Incremento del dolor
- ☐ Aumento de la rigidez
- ☐ Inestabilidad, vértigos
- ☐ Nausea
- ☐ Dolor de cabeza
- ☐ Fatiga
- ☐ Somnolencia, estupor
- ☐ Efecto adverso severo (Explicar)_____________

43. ¿Cuál es la propia apreciación actual del paciente hasta la fecha?

- ☐ Peor que nunca
- ☐ Mucho peor.
- ☐ Peor
- ☐ Ni mejor/Ni peor
- ☐ Mejorando
- ☐ Mucho mejor.
- ☐ Mejor que nunca

44. ¿Ha contactado usted, como ostéopata, con el médico de Cabecera del paciente durante el tratamiento?

- ☐ NO
- ☐ SI En caso afirmativo, causa de este contacto
  - ☐ Paciente que fue derivado a nuestra consulta
  - ☐ Para solicitar más información o para diagnóstico
  - ☐ El Médico de Cabecera ha solicitado información
  - ☐ Para solicitar la derivación a otro tratamiento
  - ☐ Para proporcionar información al Medico de Cabecera
  - ☐ Otros (especificar) _________________________

45. En esta última visita, ¿Qué se acordó para un futuro?

- ☐ Nada, el paciente fue dado de alta
- ☐ El paciente optó por volver para nueva revisión
- ☐ El paciente espera a resultados de pruebas diagnósticas.
- ☐ El paciente se deriva para proceso de Diagnóstico/tratamiento.
- ☐ Continúa con el tratamiento.
- ☐ El paciente retoma un tratamiento anterior previo a éste.
- ☐ Otro (indicar)

46. ¿Si el paciente es derivado para otro tratamiento mientras aún está en tratamiento osteopático, a dónde es derivado?

- ☐ A su Médico de Cabecera
- ☐ Otro medico consultor o especialista
- ☐ Otro facultativo complementario
- ☐ Fisioterapeuta o podólogo
- ☐ Actividad física dirigida
- ☐ Otros (indicar) ____________________________

47. ¿A qué NACIONALIDAD pertenece el paciente? (esta pregunta es opcional: el objetivo de la pregunta es clasificar y tratar a todos los grupos por igual ).

AREA DE ORIGEN

Iberoamérica:
- ☐ ECUADOR
- ☐ COLOMBIA
- ☐ ARGENTINA
- ☐ REPUBLICA DOMINICANA
- ☐ BOLIVIA
- ☐ PERU
- ☐ BRASIL

Europa Occidental
- ☐ ESPAÑOL
- ☐ REINO UNIDO
- ☐ ALEMANIA
- ☐ ITALIA
- ☐ FRANCIA
- ☐ PORTUGAL
- ☐ PAISES BAJOS

Europa del Este
- ☐ RUMANIA
- ☐ BULGARIA
- ☐ UCRANIA
- ☐ POLONIA
- ☐ RUSIA
- ☐ LITUANIA

África del Norte
- ☐ MARRUECOS
- ☐ ARGELIA

África subsahariana
- ☐ SENEGAL
- ☐ NIGERIA
- ☐ GAMBIA

Extremo Oriente
- ☐ CHINA
- ☐ FILIPINAS
- ☐ JAPON

Subcontinente Indio
- ☐ PAKISTAN
- ☐ INDIA
- ☐ BANGLADÉS

América del Norte
- ☐ EEUU

Medio Oriente
- ☐ IRAN
- ☐ SIRIA
- ☐ ISRAEL

Oceanía
- ☐ AUSTRALIA

Otros y especificar

______________________

Gracias por rellenar este formulario

# Osteopathic Standardised Data Collection*

## Declaración de la acreditación

"Esta herramienta estandarizada de toma de datos ha sido elaborada por el Consejo Nacional de Investigación osteopática (NCOR), y financiado por el Consejo General de Osteopatía (GOsC), el consejo regulador de osteopatas de UK. Los derechos de propiedad intelectual de esta herramienta estandarizada son propiedad conjunta de la NCOR y GOsC. La herramienta debe ser referenciada en el trabajo publicado como: *Moore AP, Leach CMJ, Fawkes CA. Standardised data collection tool for osteopathic practice. National Council for Osteopathic Research (UK) and General Osteopathic Council UK, 2009".*
