# Supplementary material for: Profile of osteopathic practice in Spain: results from a standardized data collection study
Source: BMC Complement Altern Med. 2018 Apr 11;18:129. doi: 10.1186/s12906-018-2190-0 (PMC5896131; doi:10.1186/s12906-018-2190-0)
Supplement: Supplementary file 4 — Professional survey. A Survey containing 20 questions covering osteopaths’ professional profile. (ZIP 172 kb) [file 12906_2018_2190_MOESM4_ESM.zip › Additional file 3 (EN)R3.pdf]

GR OstBCN
Grup de Recerca en Osteopatia de Barcelona

FORM TO BE COMPLETED BY THE OSTEOPATHY PROFESSIONAL

The osteopath must answer these 19 questions. We ask the osteopathic professional send the complete forms in the envelope that we provide in the same shipment after its completion and in the indicated terms. ***The fact of participating in the study is considered a voluntary agreement between the researcher and the osteopath.*** The data will be treated anonymously and confidentially. For any questions, contact us in the following email info@grostbcn.com.

I. PROFESSIONAL CODE Exemple: Ramón Martínez Ramos: ramara

1. Code: I__I__I__I__I__I__I   2. Age: I__I__I (years old).   3. Sex: Male ☐ Female ☐

II. FORMATION

4. Previous university studies

☐ Yes Indicate studies: ______________________ Universitiy (see university code): I__I__I__I
Year Finished studies: I__I__I__I__I

☐ No

III. OSTEOPATHY STUDIES

5. ☐ European School 6. ☐ Spanish School . ¿What school was to study? ______________________

8. Year of completion studies of osteopathy I__I__I__I__I

9. Years of experience like a Osteopath: I__I__I

10. Duration of Osteopathy studies in hours:

☐ less than 500 h
☐ between 500 h and 1000 h
☐ between 1000 h and 1500 h
☐ more than 1500 h

IV. WORKPLACE

11. Private consultation ☐ 12. Own Private Consultation ☐

13. Do you work in consultation with more osteopaths?

☐ Yes ☐1 ☐2 ☐3 ☐4 ☐more than 5
☐ No

14. You belong a multidisciplinary team as an osteopath ☐ Yes ☐ No

15. Hours / Week of clinical activity like a osteopath: ☐ between 5 and 10h ☐ betwenn 10 and 20h ☐ more than 20h

16. ¿ Do you exercise your clinical activity exclusively like a osteopath?☐ Yes ☐ No

17. Profile of the patient that you mostly visit (mark all the possibilities):

☐ Musculoskeletal
☐ Pediatric
☐ Obstetric
☐ Gynecologcal
☐ Other: ______________________

18. Do you belongs to a Register / Association of Osteopaths?

☐ ROE ☐ APREO ☐ ROP ☐ ROFE ☐ SEMMO ☐ ANAOST
☐ No
☐ Others: ______________________

19. Do you belong to a professional school?

☐ Yes: ______________________
☐ No

THANK YOU FOR ANSWERING THE QUESTIONS

Andalucía
001 Universidad de Almería
002 Universidad de Cádiz
003 Universidad de Córdoba
004 Universidad de Granada
005 Universidad de Huelva
006 Universidad Internacional de Andalucía
007 Universidad de Jaén
008 Universidad de Málaga
009 Universidad Pablo de Olavide
010 Universidad de Sevilla

Aragón
011 Universidad de Zaragoza
012 Universidad San Jorge

Canarias
013 Universidad de La Laguna
014 Universidad de Las Palmas de Gran Canaria

Cantabria
015 Universidad de Cantabria
016 Universidad Internacional Menéndez Pelayo (UIMP)

Castilla La Mancha
017 Universidad de Castilla La Mancha

Castilla y León
018 Universidad de Burgos
019 Universidad Católica de Ávila
020 Universidad Europea Miguel de Cervantes
021 IE Universidad
022 Universidad de León
023 Universidad Pontificia de Salamanca
024 Universidad de Salamanca
025 Universidad de Valladolid

Catalunya
026 Universitat Abat Oliba CEU
027 Universitat Autónoma de Barcelona
028 Universitat de Barcelona
029 Universitat de Girona
030 Universitat Internacional de Catalunya
031 Universitat de Lleida
032 Universitat Oberta de Catalunya
033 Universitat Politécnica de Catalunya
034 Universitat Pompeu Fabra
035 Universitat Ramon Llull
036 Universitat Rovira i Virgili
037 Universitat de Vic

Comunidad de Madrid
038 Universidad Alfonso X El Sabio
039 Universidad de Alcalá
040 Universidad Antonio de Nebrija
041 Universidad Autónoma de Madrid
042 Universidad Camilo José Cela
043 Universidad Carlos III de Madrid
044 Universidad Complutense de Madrid
045 Universidad a Distancia de Madrid
046 Universidad Europea de Madrid
047 Universidad Francisco de Vitoria
048 Universidad Nacional de Educación a Distancia (UNED)
049 Universidad Politécnica de Madrid
050 Universidad Pontificia de Comillas
051 Universidad Rey Juan Carlos
052 Universidad de San Pablo-CEU

Comunidad Foral de Navarra
053 Universidad de Navarra
054 Universidad Pública de Navarra

Comunitat Valenciana
055 Universitat d'Alacant / Universidad de Alicante
056 Universitat Jaume I
057 Universidad Miguel Hernández
058 Universitat Politècnica de València
059 Universitat de València
060 Universitat CEU Cardenal Herrera
061 Universidad Católica de Valencia "San Vicente Mártir"

Extremadura
062 Universidad de Extremadura

Galicia
063 Universidade da Coruña
064 Universidade de Santiago de Compostela
065 Universidad de Vigo

Illes Balears
066 Universitat de Les Illes Balears

La Rioja
067 Universidad de La Rioja
068 Universidad Internacional de la Rioja

País Vasco
069 Mondragon Unibertsitatea
070 Universidad de Deusto
071 Universidad del País Vasco / Euskal Herriko Unibertsitatea

Principado de Asturias
072 Universidad de Oviedo

Región de Murcia
073 Universidad Politécnica de Cartagena
074 Universidad Católica de San Antonio
075 Universidad de Murcia
