# Supplementary material for: Profile of osteopathic practice in Spain: results from a standardized data collection study
Source: BMC Complement Altern Med. 2018 Apr 11;18:129. doi: 10.1186/s12906-018-2190-0 (PMC5896131; doi:10.1186/s12906-018-2190-0)
Supplement: Supplementary file 4 — Professional survey. A Survey containing 20 questions covering osteopaths’ professional profile. (ZIP 172 kb) [file 12906_2018_2190_MOESM4_ESM.zip › Additional file 3R3.pdf]

Perfil de la práctica osteopática en España: Estudio transversal.

A profile of Osteophatic practice in Spain: a cross sectional survey.

GROstBCN
Grup de Recerca en Osteopatia de Barcelona

FORMULARIO A CUMPLIMENTAR POR EL PROFESIONAL OSTEOPATA

Se solicita a cada osteópata responder a estas 19 preguntas. Rogamos que cada profesional osteópata envíe los formularios completos en el sobre que facilitamos en el propio envío con la máxima celeridad posible después de su cumplimentación y en los términos indicados. ***El hecho de participar en el estudio se considera como un acuerdo voluntario y consentido entre investigador y osteópata.*** Los datos serán tratados de forma anónima y con carácter confidencial. Para cualquier duda diríjase a nosotros a través del correo info@grostbcn.com.

I. CÓDIGO PROFESIONAL Ejemplo: Ramón Martínez Ramos: ramara

1. Código: I__I__I__I__I__I__I 2. Edad: I__I__I (años). 3. Sexo: Masculino ☐ Femenino ☐

II. FORMACIÓN

4. Estudios previos universitarios

☐ Si Indicar cuál: ______________________ Universidad (ver código en reverso): I__I__I__I
Año finalización estudios: I__I__I__I__I

☐ No

III. ESTUDIOS DE OSTEOPATIA

5. ☐ Escuela Europea 6. ☐ Escuela Española . ¿En qué escuela estudió? ______________________

8. ¿En qué año finalizó los estudios de Osteopatía? I__I__I__I__I

9. Años de experiencia como Osteópata: I__I__I

10. Duración de los estudios de Osteopatía en horas:
☐ menos de 500 h
☐ entre 500 h y 1000 h
☐ entre 1000 h y 1500 h
☐ más de 1500 h

IV. LUGAR DE TRABAJO

11. Consulta privada ☐ 12. Consulta privada propia ☐
13. ¿Trabaja en consulta con más osteópatas?
☐ Si ☐1 ☐2 ☐3 ☐4 ☐más de 5
☐ No
14. Forma usted parte de un equipo multidisciplinar como osteópata ☐
15. Otros ______________________
16. Horas empleadas a la semana: ☐ entre 5 y 10h ☐ entre 10 y 20h ☐ más de 20h
17. ¿Ejerce su actividad clínica exclusivamente como osteópata? ☐ Si ☐ No
18. Perfil del paciente que mayoritariamente visita (marque todas las posibilidades):
☐ Musculoesquelético
☐ Pediátrico
☐ Obstétrico
☐ Ginecológico
☐ Otros: ______________________

19. ¿Pertenece a algún Registro/Asociación de Osteópatas?
☐ Si ☐ROE ☐APREO ☐ROP ☐ROFE ☐SEMMO ☐ANAOST
☐ No
20. ¿Pertenece a algún colegio Profesional)
☐ Si Indique cuál: ______________________
☐ No

GRACIAS POR RESPONDER A LAS PREGUNTAS

Andalucía
001 Universidad de Almería
002 Universidad de Cádiz
003 Universidad de Córdoba
004 Universidad de Granada
005 Universidad de Huelva
006 Universidad Internacional de Andalucía
007 Universidad de Jaén
008 Universidad de Málaga
009 Universidad Pablo de Olavide
010 Universidad de Sevilla

Aragón
011 Universidad de Zaragoza
012 Universidad San Jorge

Canarias
013 Universidad de La Laguna
014 Universidad de Las Palmas de Gran Canaria

Cantabria
015 Universidad de Cantabria
016 Universidad Internacional Menéndez Pelayo (UIMP)

Castilla La Mancha
017 Universidad de Castilla La Mancha

Castilla y León
018 Universidad de Burgos
019 Universidad Católica de Ávila
020 Universidad Europea Miguel de Cervantes
021 IE Universidad
022 Universidad de León
023 Universidad Pontificia de Salamanca
024 Universidad de Salamanca
025 Universidad de Valladolid

Catalunya
026 Universitat Abat Oliba CEU
027 Universitat Autónoma de Barcelona
028 Universitat de Barcelona
029 Universitat de Girona
030 Universitat Internacional de Catalunya
031 Universitat de Lleida
032 Universitat Oberta de Catalunya
033 Universitat Politécnica de Catalunya
034 Universitat Pompeu Fabra
035 Universitat Ramon Llull
036 Universitat Rovira i Virgili
037 Universitat de Vic

Comunidad de Madrid
038 Universidad Alfonso X El Sabio
039 Universidad de Alcalá
040 Universidad Antonio de Nebrija
041 Universidad Autónoma de Madrid
042 Universidad Camilo José Cela
043 Universidad Carlos III de Madrid
044 Universidad Complutense de Madrid
045 Universidad a Distancia de Madrid
046 Universidad Europea de Madrid
047 Universidad Francisco de Vitoria
048 Universidad Nacional de Educación a Distancia (UNED)
049 Universidad Politécnica de Madrid
050 Universidad Pontificia de Comillas
051 Universidad Rey Juan Carlos
052 Universidad de San Pablo-CEU

Comunidad Foral de Navarra
053 Universidad de Navarra
054 Universidad Pública de Navarra

Comunitat Valenciana
055 Universitat d'Alacant / Universidad de Alicante
056 Universitat Jaume I
057 Universidad Miguel Hernández
058 Universitat Politècnica de València
059 Universitat de València
060 Universitat CEU Cardenal Herrera
061 Universidad Católica de Valencia "San Vicente Mártir"

Extremadura
062 Universidad de Extremadura

Galicia
063 Universidade da Coruña
064 Universidade de Santiago de Compostela
065 Universidad de Vigo

Illes Balears
066 Universitat de Les Illes Balears

La Rioja
067 Universidad de La Rioja
068 Universidad Internacional de la Rioja

País Vasco
069 Mondragon Unibertsitatea
070 Universidad de Deusto
071 Universidad del País Vasco / Euskal Herriko Unibertsitatea

Principado de Asturias
072 Universidad de Oviedo

Región de Murcia
073 Universidad Politécnica de Cartagena
074 Universidad Católica de San Antonio
075 Universidad de Murcia
